# CLDN6 inhibits breast cancer growth and metastasis through SREBP1-mediated RAS palmitoylation

**DOI:** 10.1186/s11658-024-00629-y

**Published:** 2024-08-21

**Authors:** Qiu Jin, Da Qi, Mingzi Zhang, Huinan Qu, Yuan Dong, Minghao Sun, Chengshi Quan

**Affiliations:** 1 The Key Laboratory of Pathobiology, Ministry of Education, College of Basic Medical Sciences, Jilin University, 126 Xinmin Avenue, Changchun, 130021 Jilin China; 2 The Zilkha Neurogenetic Institute, Department of Physiology and Neuroscience Keck School of Medicine of the University of Southern California, 1501 San Pablo Street, Los Angeles, 90033 CA US; 3 Department of Histology and Embryology, College of Basic Medical Sciences, Jilin University, 126 Xinmin Avenue, Changchun, 130021 Jilin China

**Keywords:** CLDN6, SREBP1, Fatty acid, RAS, Palmitoylation, Breast cancer

## Abstract

**Background:**

Breast cancer (BC) ranks as the third most fatal malignant tumor worldwide, with a strong reliance on fatty acid metabolism. CLDN6, a candidate BC suppressor gene, was previously identified as a regulator of fatty acid biosynthesis; however, the underlying mechanism remains elusive. In this research, we aim to clarify the specific mechanism through which CLDN6 modulates fatty acid anabolism and its impact on BC growth and metastasis.

**Methods:**

Cell function assays, tumor xenograft mouse models, and lung metastasis mouse models were conducted to evaluate BC growth and metastasis. Human palmitic acid assay, triglyceride assay, Nile red staining, and oil red O staining were employed to investigate fatty acid anabolism. Reverse transcription polymerase chain reaction (RT–PCR), western blot, immunohistochemistry (IHC) assay, nuclear fractionation, immunofluorescence (IF), immunoprecipitation and acyl–biotin exchange (IP-ABE), chromatin immunoprecipitation (ChIP), dual luciferase reporter assay, and co-immunoprecipitation (Co-IP) were applied to elucidate the underlying molecular mechanism. Moreover, tissue microarrays of BC were analyzed to explore the clinical implications.

**Results:**

We identified that CLDN6 inhibited BC growth and metastasis by impeding RAS palmitoylation both in vitro and in vivo. We proposed a unique theory suggesting that CLDN6 suppressed RAS palmitoylation through SREBP1-modulated de novo palmitic acid synthesis. Mechanistically, CLDN6 interacted with MAGI2 to prevent KLF5 from entering the nucleus, thereby restraining SREBF1 transcription. The downregulation of SREBP1 reduced de novo palmitic acid synthesis, hindering RAS palmitoylation and subsequent endosomal sorting complex required for transport (ESCRT)-mediated plasma membrane localization required for RAS oncogenic activation. Besides, targeting inhibition of RAS palmitoylation synergized with CLDN6 to repress BC progression.

**Conclusions:**

Our findings provide compelling evidence that CLDN6 suppresses the palmitic acid-induced RAS palmitoylation through the MAGI2/KLF5/SREBP1 axis, thereby impeding BC malignant progression. These results propose a new insight that monitoring CLDN6 expression alongside targeting inhibition of palmitic acid-mediated palmitoylation could be a viable strategy for treating oncogenic RAS-driven BC.

**Graphical Abstract:**

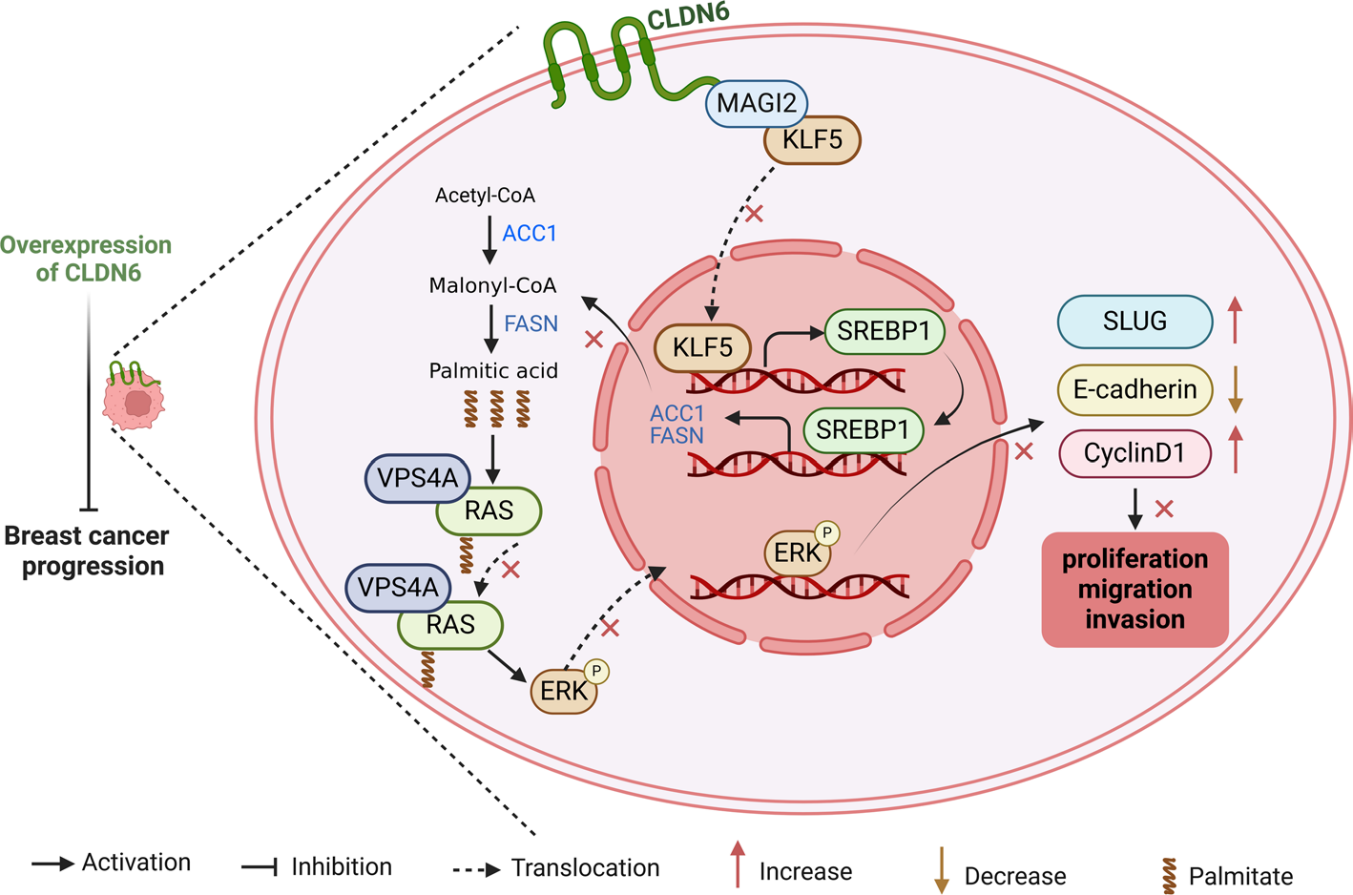

**Supplementary Information:**

The online version contains supplementary material available at 10.1186/s11658-024-00629-y.

## Background

BC is the most commonly detected cancer and the primary reason for deaths related to cancer in women worldwide [1, 2], mainly due to recurrence [3] and metastasis [4]. Hence, there is an urgent necessity to investigate innovative molecular biological mechanisms that regulate BC progression, leading to the development of highly targeted therapies.

Reprogramming of fatty acid metabolism is an important factor contributing to BC malignant procession [5–7]. In addition to providing the energy and biofilm necessary for tumor survival and development, fatty acid metabolism can also influence a variety of epigenetic modifications [8, 9]. Representatively, palmitoylation is a widespread post-transcriptional modification closely linked to fatty acid anabolism. It utilizes palmitic acid as a substrate, binding palmitate to specific cysteine residues of target proteins through thioester bonding [10]. Palmitoylation affects tumor growth and metastasis by manipulating protein subcellular localization, oncogenic activation, and protein stability of key oncogenic proteins [11], including RAS [12], PD-L1 [13], and so on. As a member of low-molecular-weight GTP-binding proteins, RAS requires palmitoylation for its localization to the cell membrane and subsequent activation to drive malignant tumor progression [14]. However, it remains unclear whether fatty acid anabolism can influence BC progression by manipulating RAS palmitoylation.

CLDN6, a quintessential tight junction protein, promotes the establishment of the epithelial barrier and fence [15]. The PDZ-binding motif (PBM) at CLDN6’s carboxyl terminal facilitates its interaction with proteins containing PDZ domains, thereby mediating the transmission of intracellular signals [16]. Previously, we confirmed the role of CLDN6 as a tumor suppressor of BC for the first time [17]. Remarkably, nontargeted metabolomics revealed that overexpression of CLDN6 resulted in significant enrichment of differential metabolites within the fatty acid biosynthesis pathway [18]. Moreover, our previous RNA sequencing (RNA-seq) analysis of BC cells demonstrated that SREBP1 expression was downregulated following CLDN6 overexpression [19]. Serving as a fundamental transcription factor that facilitates de novo fatty acid synthesis, SREBP1, encoded by SREBF1, enhances fatty acid synthesis in tumor cells by upregulating pivotal enzymes associated with lipogenesis, such as ACC1, FASN, and SCD1 [20]. However, whether CLDN6 can affect palmitoylation in BC through de novo fatty acid synthesis has never been reported.

Our study elucidated for the first time that CLDN6 suppressed palmitic acid-induced RAS palmitoylation through de novo fatty acid synthesis, leading to the inhibition of BC growth and metastasis. Mechanistically, CLDN6 interacted with MAGI2 to transcriptionally inhibit SREBP1 expression by repressing KLF5 nuclear translocation, thereby impairing palmitic acid-mediated RAS palmitoylation and, ultimately, repressing the carcinogenic activation of RAS. These findings unveiled a novel mechanism through which CLDN6 regulated BC progression, offering a new experimental foundation for the development of clinical targeted therapies.

## Materials and methods

### Cell culture and transfection

The MCF-7, MDA-MB-231, and HEK293T cells were cultured in Dulbecco’s modified Eagle medium (DMEM)/high glucose (Meilune, Dalian, China) supplemented with 10% fetal bovine serum (FBS; Gibco, Carlsbad, CA) and 1% penicillin–streptomycin (Meilune, Dalian, China). The cells were incubated in a temperature-controlled incubator at 37 ℃ with a 5% CO_2_ environment. The culture medium was renewed daily. Regular screenings using the Mycoplasma PCR Detection Kit (Meilune, Dalian, China) were conducted to ensure the absence of mycoplasma contamination. The MCF-7 (cat. no. ZQ0071), MDA-MB-231 (cat. no. ZQ0118), and HEK293T (cat. no. ZQ0033) cell lines were procured from Zhong Qiao Xin Zhou Biotechnology (Shanghai, China). The transfection procedures were conducted according to the methodology outlined in the study of Yang et al. [21]. The plasmids utilized in this study were exclusively sourced from PPL Genebio Technology (Nanjing, China). Fatostatin (HY-14452) and C75 (HY-12364) were purchased from MCE (USA). 2BP (E0120) was obtained from Selleck (China) and palmitic acid (SYSJ-KJ003) was purchased from Kunchuang biotechnology (China).

### Reverse transcription polymerase chain reaction (RT–PCR)

The RT–PCR procedure was performed in accordance with the protocol that had been previously outlined [22]. The primers utilized in this research were custom-synthesized by Sangon (Shanghai, China) and are available for reference in Supplementary Materials.

### Western blot

Western blot was conducted following the procedure previously outlined [23]. Supplementary Materials provide a list of antibodies utilized in this study.

### Cell counting kit 8 assay (CCK-8)

The evaluation of cellular viability was conducted by employing CCK-8 (Meilune, Dalian, China). A total of 1 × 10^3^ BC cells were distributed into 96-well plates and then allowed to incubate for a duration of 24 h. A CCK-8 solution diluted at a ratio of 1:9 in DMEM was introduced to per well and then incubated at a temperature of 37 ℃ for a duration of 1 h under a 5% CO_2_ environment. The sample’s absorbance was assessed at the wavelength of 450 nm.

### Colony formation assay

The BC cells were cultured in six-well plates with a seeding density of 500 cells per well and maintained for a period of 10 d. After fixation with 4% paraformaldehyde (Solarbio, Beijing, China), the cells were stained using crystal violet solution (Solarbio, Beijing, China). Finally, photography was employed to capture the samples.

### Cell cycle analysis

Cell cycle progression was analyzed employing the Cell Cycle and Apoptosis Analysis Kit (Meilun, Dalian, China) in accordance with the manufacturer’s instructions followed by flow cytometry. In brief, cells were collected and preserved using a solution containing 75% ethanol at a temperature of 4 ℃ for an overnight duration. Following centrifugation, the staining solution was applied to the cells for a period of 30 min. Subsequently, flow cytometry analysis was conducted by utilizing a flow cytometer (FACSAria, Biosciences, USA).

### Wound healing assay

The BC cells were grown in six-well dishes and then scratched after cell attachment. Subsequently, the FBS-free DMEM medium was used to incubate the cells. To assess the alteration of the wounded area, photographs were captured at the start and after 24 and 48 h.

### Transwell migration and invasion assays

Transwell chambers (Corning, Lowell, MA) were employed to evaluate the BC cells’ migratory and invasive potential. To assess cellular migration, we introduced 2.5 × 10^4^ cells into the upper chamber, while the lower chamber was filled with DMEM medium enriched with 10% FBS. For the purpose of invasion analysis, a layer of Matrigel (Corning, Lowell, MA) was applied onto the chamber surface. The Matrigel was diluted at a ratio of 1:8 with DMEM. The subsequent procedures are consistent with those employed in the migration assay. Following a 24-h incubation period, the cells underwent fixation by treatment with a 4% paraformaldehyde solution and were then subjected to staining using crystal violet. The quantification of cells that migrated and invaded was performed in three fields chosen at random.

### Nile red staining

After being seeded into 24-well plates, BC cells were subjected to fixation using a 4% paraformaldehyde solution for a period of 15 min. Following this, BC cells or frozen sections of the transplanted tumor were subjected to staining using Nile red (Meilun, Dalian, China) for 20 min and 4′,6-diamidino-2-phenylindole (DAPI, Meilun, Dalian, China) for 5 min at room temperature. Subsequently, fluorescence microscopy (Olympus, Japan) was employed to capture images.

### Oil red O staining

The oil red O Stain Kit (Solarbio, Beijing, China) was employed for the detection of neutral lipid content. The frozen sections of xenograft tumor tissue were allowed to thaw at ambient temperature for a duration of 20 min, followed by sequential rinsing with distilled water and 60% isopropanol. Subsequently, the sections were subjected to staining using oil red O working solution in a light-restricted environment for a period of 15 min. After hematoxylin staining for 5 min, glycerin gelatin (Solarbio, Beijing, China) was employed for sealing the slices.

### Human palmitic acid assay

The assay for human palmitic acid was conducted in accordance with the instructions of Human Palmitic Acid ELISA Kit (Youxuan, Shanghai, China). The measurement of palmitic acid content was performed utilizing a microplate reader that operated at the wavelength of 450 nm.

### Triglyceride assay

The cell samples were initially obtained and subsequently subjected to lysis. The triglyceride reagent kit (Jiancheng, Nanjing, China) was employed to ascertain the concentration of triglycerides based on the instructions provided by the manufacturer. The BCA Kit (Meilun, Dalian, China) was utilized to assess the total protein concentration, and the triglyceride levels were measured at 510 nm by microplate reader.

### Nuclear fractionation

After the collection of 3 × 10^6^ cells according to the guidelines provided by the manufacturer, we employed a Mammalian Nuclear and Cytoplasmic Protein Extraction Kit (TransGen Biotech, Beijing, China) for the separation of proteins in the nucleus and cytoplasm.

### Immunohistochemistry (IHC) assay

The tissue microarray of human BC (cat. no. HBreD070CS02) was acquired from Outdo Biotech Company (Shanghai, China). The IHC procedure was conducted following the guidelines specified by the IHC Kit (MXB Biotechnology, Fuzhou, China). The IHC scores were determined by considering both the positive intensity (0 indicating absence, 1 representing low, 2 denoting moderate, and 3 signifying high) and the ratio of cells exhibiting a positive response (1 for ≤ 25%, 2 for 26–50%, 3 for 51–75%, and 4 for ≥ 75%). Supplementary Materials contain the antibodies that were employed.

### Chromatin immunoprecipitation (ChIP) assay

The ChIP procedure was conducted in accordance with the previously delineated methodology [24]. The primers sequences can be found in Supplementary Materials.

### Immunoprecipitation-acyl-biotin exchange (IP-ABE)

The IP-ABE was performed following the recommendations of the IP-ABE Palmitoylation Kit for western blot (Aimsmass, Shanghai, China). Briefly, cells were lysed and subsequently centrifuged prior to overnight immunoprecipitation with agarose beads and specific antibodies. After three washes, the precipitates were divided equally into two portions: one portion served as the HAM- control, while the other half underwent HAM + treatment at room temperature for 60 min. The precipitate was gently washed and subsequently incubated with BMCC–biotin buffer for 60 min. Following washing steps, samples were analyzed through western blot analysis, where palmitoylated RAS or HRAS was detected using HRP-conjugated streptavidin provided in the kit. The antibodies employed in this investigation included RAS (ab52939, Abcam, US) and HRAS (18295–1-AP, Proteintech, China).

### Animal experiments

For the mouse models of tumor xenografts, female BALB/c–nu mice (a weight range of 16–20 g and 4 weeks old) were procured from the animal supplier Huafukang Company (Beijing, China). The subcutaneous injection of 100 μL PBS containing 0.5 × 10^6^ BC cells was administered to nude mice. The intraperitoneal injection of 2BP (at a dosage of 30 mg/kg body weight) was administered every other day for 2 weeks after 1 week of implantation. By determining the length (*L*) and width (*W*), the measurements of tumor volume (*V*) were obtained and determined by employing the equation *V* = 0.5 × *L* × *W*^2^.

For the BC lung metastasis models, the BALB/c-nu female nude mice were injected with 100 μL PBS containing 1 × 10^6^ BC cells via the tail vein. After 1 week, the intraperitoneal injection of 2BP (at a dosage of 30 mg/kg body weight) was administered every other day. After a duration of 3 weeks, mice were administered an intraperitoneal injection of d-luciferin. Subsequently, luciferase imaging was conducted after a time interval of 10 min. The detection of metastasis ability was accomplished through the utilization of hematoxylin and eosin (H&E) staining.

### Immunofluorescence (IF)

The IF assay was conducted following the established protocol [25]. Supplementary Materials contain a list of antibodies employed in the IF analysis.

### Dual luciferase reporter assay

In accordance with the preestablished protocol, the dual luciferase reporter assay was performed [22]. The Mut plasmid contains the AAAATAAAAT base sequence, which was mutated from the GGGGCGGGGGC base sequence of the KLF5 binding site in the SREBF1 promoter region.

### Co-immunoprecipitation (Co‑IP) assay

To identify protein–protein interactions, we conducted the Co-IP assay following the methods outlined in a prior study [26]. Supplementary Materials contain the antibodies that were employed.

### Statistical analysis

The study’s statistical analysis was exclusively conducted using GraphPad Prism (GraphPad, CA). The final statistical results were reported as the average value with the standard deviation (SD) calculated from at least three separate trials. The normality of the samples was evaluated using Shapiro–Wilk test (*n* < 50) or Kolmogorov–Smirnov test (*n* > 50). Unpaired Student’s *t*-test and Mann–Whitney *U* test were respectively employed for the comparison of normal and non-normal variables between two groups. A one-way analysis of variance (ANOVA) and Kruskal–Wallis test were respectively utilized as parametric and nonparametric approaches to compare more than two groups. The association between clinical parameters and protein expression was assessed using a two-sided chi-square test. The examination of protein correlations involved the computation of Pearson’s correlation coefficients. A significance level of *P* < 0.05 was utilized to ascertain the statistical significance.

## Results

### CLDN6 inhibits BC growth and metastasis through fatty acid biosynthesis

In previous study, we observed a low expression of CLDN6 in BC [27]. To elucidate the biological role of CLDN6, we generated BC cell lines stably overexpressing GFP-fused CLDN6 (Supplementary Fig. 1A). Notably, CLDN6 overexpression significantly reduced cell viability (Supplementary Fig. 1B) and clonogenicity (Supplementary Fig. 1C) while induced G1/S phase arrest across different BC cell lines (Supplementary Fig. 1D). Consistently, tumors derived from MDA-MB-231/CLDN6 cells demonstrated reduced growth rates and lower weights (Supplementary Fig. 1E–G). Moreover, the suppressive impact of CLDN6 on the migratory and invasive capabilities of BC cells was demonstrated (Supplementary Fig. 1H–I), consistent with our previous findings that overexpression of CLDN6 restrained BC metastasis in vivo [22]. These results strongly supported CLDN6’s inhibitory role in BC progression, prompting us to further investigate its underlying mechanism.

**Fig. 1:**
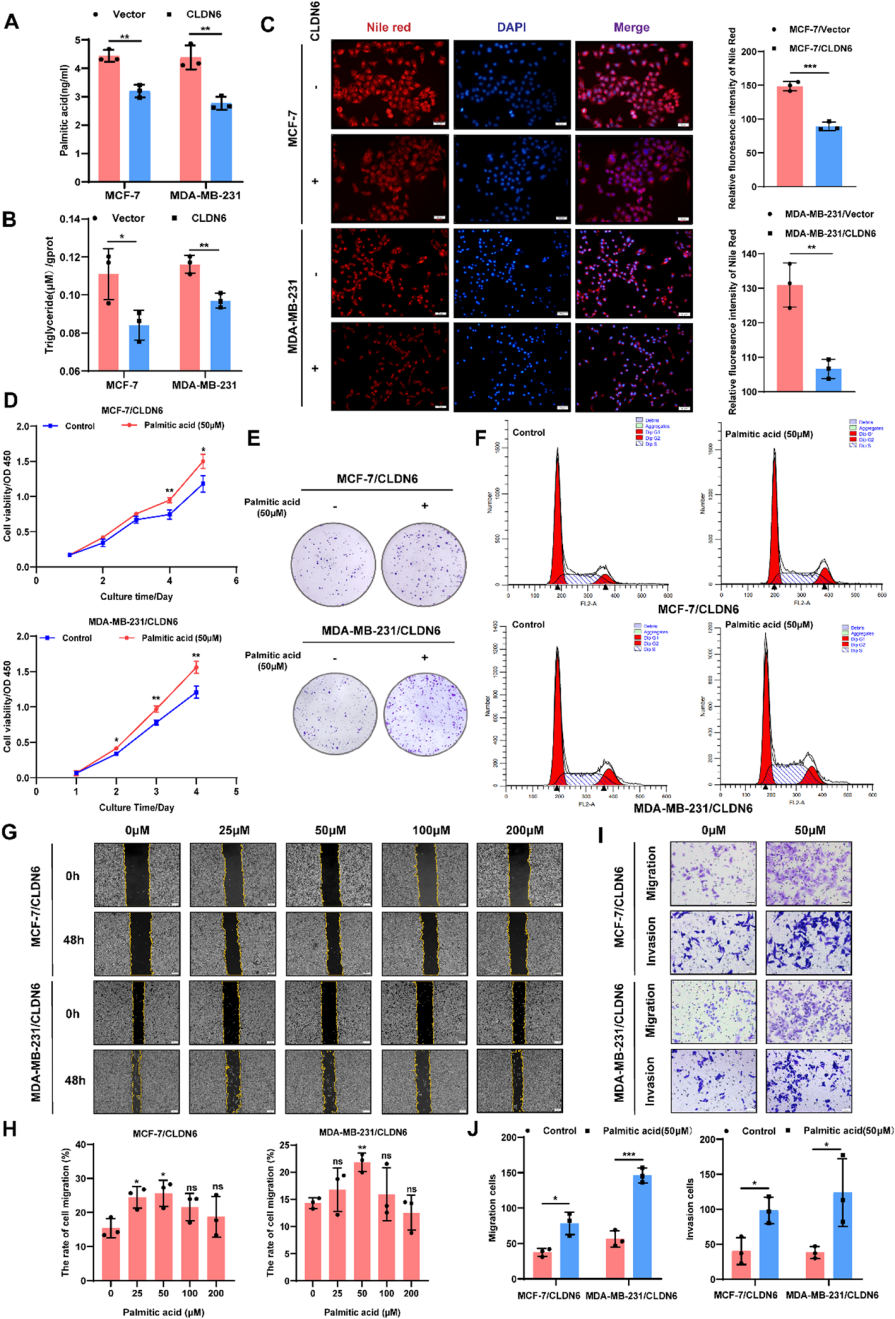
Inhibitory effects of CLDN6 on fatty acid anabolism-induced BC progression. **A**, **B** Palmitic acid and triglyceride content in BC cells with CLDN6 overexpression (*n* = 3). **C** Nile red staining detected the effect of CLDN6 on neutral lipid content. Scale bar, 50 μm. **D**-**F** The impact of palmitic acid on cell viability (**D**), the clonogenicity (**E**) and cell cycle progression (**F**) in BC cells with CLDN6 overexpression. **G**–**J** The palmitic acid-induced alterations in cellular migratory and invasive capacity of BC cells with CLDN6 overexpression (*n* = 3). Scale bar, 200 μm (**G**) and 50 μm (**I**). *P < 0.05, **P < 0.01, ***P < 0.001 denoted the presence of statistically significant disparities

Notably, the untargeted metabolomics analysis revealed that differential metabolites were predominantly enriched in the fatty acid biosynthesis pathway subsequent to CLDN6 overexpression in BC cells. Considering the significant importance of fatty acid anabolism in BC, we investigated the regulatory function of CLDN6 in this process. Our findings indicated that CLDN6 overexpression significantly reduced palmitic acid content (Fig. 1A). Furthermore, both triglycerides (Fig. 1B) and neutral lipids (Fig. 1C), which are synthesized from fatty acid substrates, exhibited a substantial reduction. Oil red O and Nile red staining also confirmed a significant decline in neutral lipids within BC xenograft tissue upon overexpression of CLDN6 (Supplementary Fig. 1J).

Interestingly, the restrictive effects of CLDN6 on cell viability (Fig. 1D), clonogenicity (Fig. 1E and Supplementary Fig. 1K) and cell cycle progression (Fig. 1F and Supplementary Fig. 1L) were partially ameliorated with exogenous palmitic acid supplementation. Meanwhile, palmitic acid compromised the suppressive impact of CLDN6 on BC cells migration and invasion (Fig. 1G–J). Collectively, these findings suggested that CLDN6 repressed BC growth and metastasis through inhibiting fatty acid biosynthesis.

### CLDN6 inhibits de novo fatty acid synthesis through SREBP1

Next, we asked which genes, altered in BC cells overexpressing CLDN6, could be responsible for the substantial fatty acid reduction. To address this query, RNA-seq analysis was employed [19], revealing a noticeable downregulation of SREBF1 expression in BC cells with CLDN6 overexpression (Supplementary Fig. 2A). Encoded by SREBF1, SREBP1 exhibited a significant upregulation in primary and metastatic BC tissues in TNMplot database (https://tnmplot.com/analysis) (Supplementary Fig. 2B). Furthermore, the Timer database (http://timer.cistrome.org/) elucidated a negative correlation between CLDN6 and SREBF1, as well as its downstream targets FASN, ACACA, and SCD, in BC (Supplementary Fig. 2C). It suggested that the disruptive impact of CLDN6 on fatty acid anabolism may rely on its regulation of SREBP1.

**Fig. 2:**
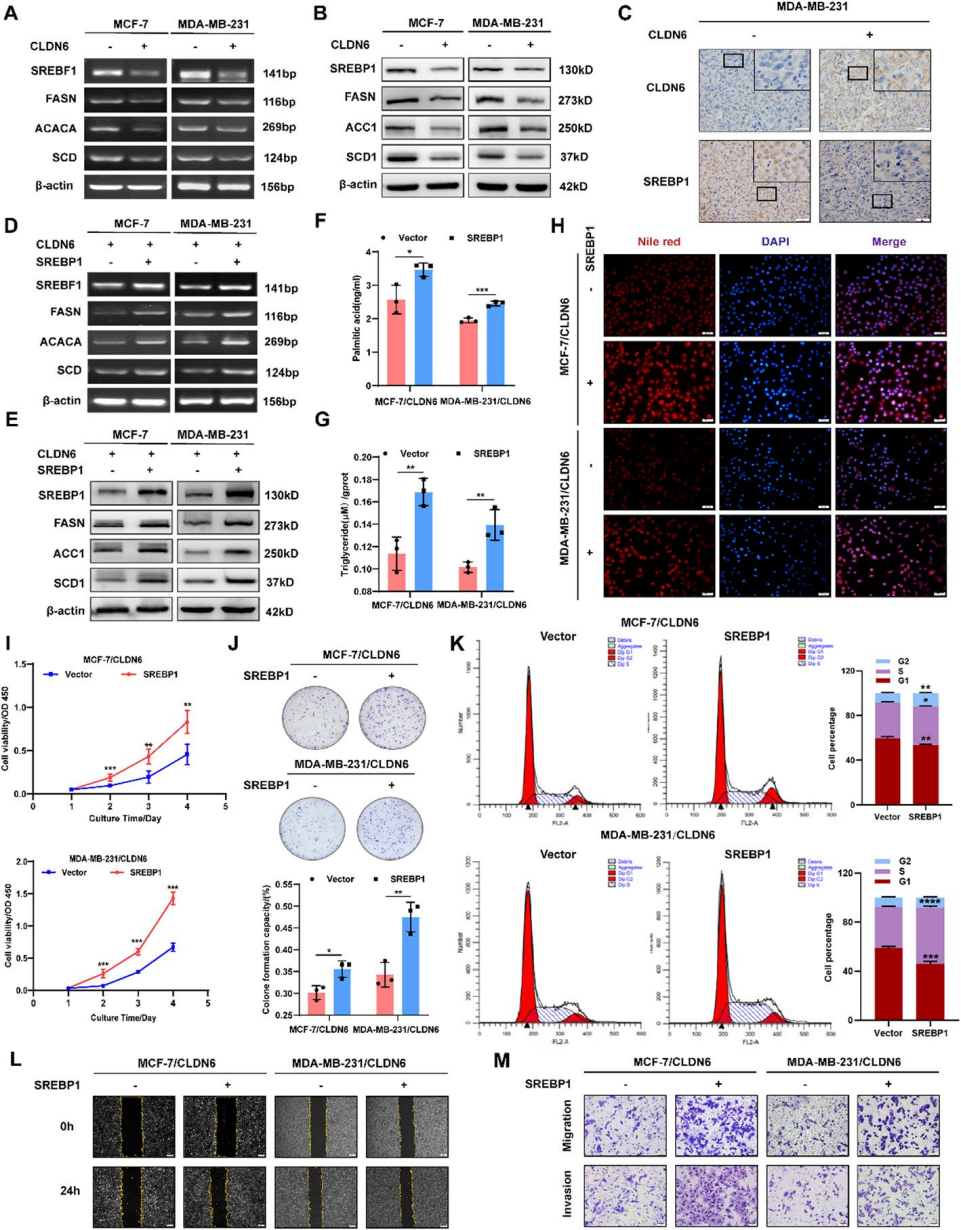
CLDN6 exerts an inhibitory effect on de novo fatty acid synthesis by downregulating SREBP1 expression. **A**, **B** The effect of CLDN6 overexpression on SREBP1, FASN, ACC1, and SCD1 expression at mRNA and protein levels in BC cells (*n* = 3). **C** Visualization of the expression levels of CLDN6 and SREBP1 in xenograft tumor tissues. Scale bar, 20 μm. **D**-**E** The effect of SREBP1 overexpression on key lipogenic enzymes expression at mRNA and protein levels in CLDN6-overexpressing BC cells (*n* = 3). **F**–**H** The impact of SREBP1 overexpression on palmitic acid, triglyceride and neutral lipid in BC cells with CLDN6 overexpression (*n* = 3). Scale bar, 50 μm. **I**–**K** The impact of SREBP1 overexpression on cell viability (**I**), clonogenicity (**J**), and cell cycle progression (**K**) of BC cells with CLDN6 overexpression. **L**, **M** The SREBP1 overexpression-induced alterations in cellular migratory (**L**) and invasive (**M**) capacity of BC cells with CLDN6 overexpression (*n* = 3). Scale bar, 200 μm (**L**) and 50 μm (**M**). *P < 0.05, **P < 0.01, ***P < 0.001 denoted the presence of statistically significant disparities

Herein, we validated that CLDN6 overexpression significantly restrained the expression of SREBP1, FASN, ACC1, and SCD1 at both mRNA and protein levels (Fig. 2A, B and Supplementary Fig. 2D–E). IHC of xenograft tumor tissues from MDA-MB-231/CLDN6 cells also displayed decreased SREBP1 expression (Fig. 2C). Furthermore, the fluorescence intensity of SREBP1 in BC cells was substantially reduced upon CLDN6 modulation (Supplementary Fig. 2F); however, the extent to which this regulation by CLDN6 affected de novo fatty acid synthesis through modulating SREBP1 necessitates further investigation.

Consequently, we evaluated whether CLDN6 inhibited malignant phenotype of BC cells through SREBP1-mediated de novo fatty acid synthesis. To accomplish this objective, SREBP1 was overexpressed and the transfection efficiency was confirmed (Fig. 2D, E, Fig. S3A-B). Notably, we found that upregulation of SREBP1 led to an elevation in the expression of FASN, ACC1, and SCD1 at both mRNA and protein levels (Fig. 2D, E, Fig. S3A-B) while significantly attenuating the suppressive effect exerted by CLDN6 on palmitic acid content (Fig. 2F), triglycerides accumulation (Fig. 2G), and neutral lipid production (Fig. 2H, Fig. S3C). Meanwhile, overexpression of SREBP1 enhanced the cell viability (Fig. 2I) and clonogenicity (Fig. 2J) while reducing the population of cells that undergo arrest during the G1 phase (Fig. 2K). SREBP1 prominently promoted the migratory and invasive capabilities of MCF-7/CLDN6 and MDA-MB-231/CLDN6 cells (Fig. 2L–M and Supplementary Fig. 3D–F). However, treatment with Fatostatin, a targeted inhibitor of SREBP1, impeded the generation of palmitic acid (Supplementary Fig. 4A), triglyceride (Supplementary Fig. 4B), and neutral lipid (Supplementary Fig. 4C). Further experiments verified that Fatostatin effectively reversed the stimulatory effect of SREBP1 on the proliferative capacity (Supplementary Fig. 4D, E) and the migratory and invasive properties of BC cells (Supplementary Fig. 4F–J). Our findings indicated that CLDN6 exerted a suppressive impact on the regulation of BC cell proliferation, migration, and invasion through its involvement in SREBP1-mediated de novo fatty acid synthesis.

### CLDN6 relies on SREBP1-mediated palmitic acid synthesis to inhibit RAS palmitoylation

**Fig. 3:**
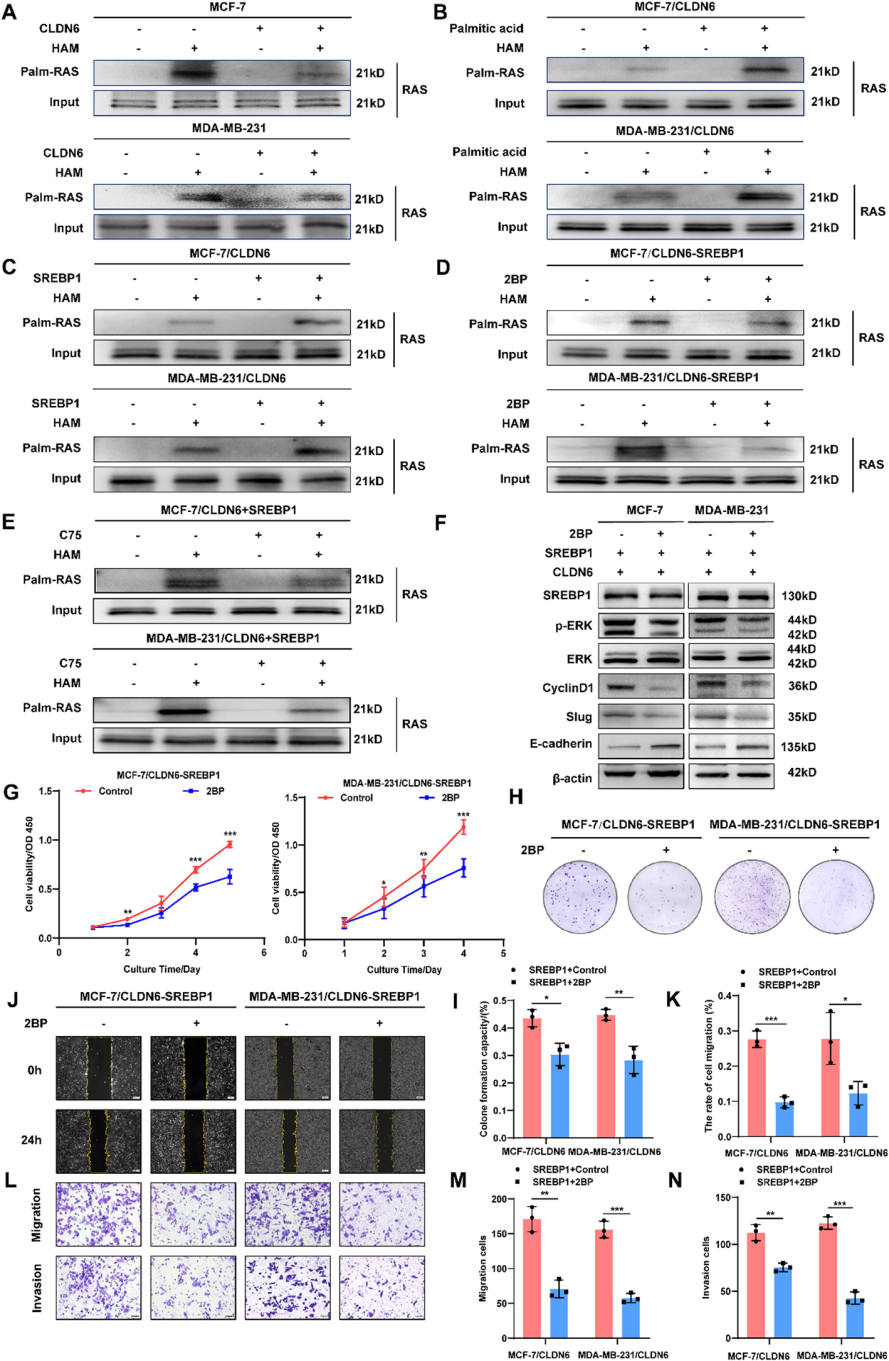
CLDN6 governs de novo palmitic acid synthesis to suppress RAS palmitoylation. **A**–**E** The alterations of RAS palmitoylation in BC cells with CLDN6 overexpression (**A**), palmitic acid treatment (**B**), SREBP1 overexpression (**C**), 2BP treatment (**D**), and C75 treatment (**E**). **F** Western blot of the protein expression in RAS/ERK signaling pathway (*n* = 3). **G**-**N** The promotion of SREBP1 on cell viability (**G**), clonogenicity (**H**, **I**), and migratory and invasive (**J**–**N**) capacity of BC cells was reversed by 2BP (*n* = 3). Scale bar, 200 μm (**J**) and 50 μm (**L**). *P < 0.05, **P < 0.01, ***P < 0.001 denoted the presence of statistically significant disparities

Our previous findings suggested that CLDN6 induced the inactivation of RAS/ERK signaling pathway [28]. Given that palmitoylation is crucial for RAS carcinogenic activation and palmitic acid rescued the repressive effects of CLDN6 on BC cell, we hypothesized that CLDN6 might hinder BC malignant progression by manipulating RAS palmitoylation. As expected, a noticeable decrease in RAS palmitoylation was detected in BC cells overexpressing CLDN6 (Fig. 3A). Following treatment with exogenous palmitic acid, CLDN6 failed to suppress the RAS palmitoylation (Fig. 3B). The results confirmed that CLDN6 inhibited RAS palmitoylation by regulating palmitic acid; however, it was worth exploring whether the inhibition of CLDN6 on palmitic acid-mediated RAS palmitoylation was conducted by SREBP1. Compared with the control group, SREBP1 overexpression notably enhanced the RAS palmitoylation (Fig. 3C). More importantly, treatment with palmitoylation inhibitor 2BP or the FASN-targeting inhibitor C75 effectively abolished the enhanced RAS palmitoylation induced by SREBP1 overexpression (Fig. 3D, E). Intriguingly, we found that CLDN6 exhibited no discernible effect on the expression of ZDHHC9 (Supplementary Fig. 5A), a palmitoyl transferase that specifically mediates RAS palmitoylation [14].

Meanwhile, treatment with 2-BP led to reduced phosphorylation levels of ERK within the RAS signaling pathway and decreased expression of downstream target proteins, namely cyclin D1, Slug, and E-cadherin. However, it did not have a significant effect on the expression of SREBP1 (Fig. 3F and Supplementary Fig. 5B). Correspondingly, we observed that 2-BP suppressed cell viability (Fig. 3G), clonogenic potential (Fig. 3H, I) and migration and invasion capabilities (Fig. 3J–N) of BC cells overexpressing SREBP1. Taken together, these findings suggested that CLDN6's inhibition of RAS palmitoylation depended on SREBP1-mediated de novo palmitic acid synthesis rather than the action of traditional palmitoyl transferase.

### CLDN6 relies on VPS4A to regulate the plasma membrane localization of palmitoylated RAS

**Fig. 4:**
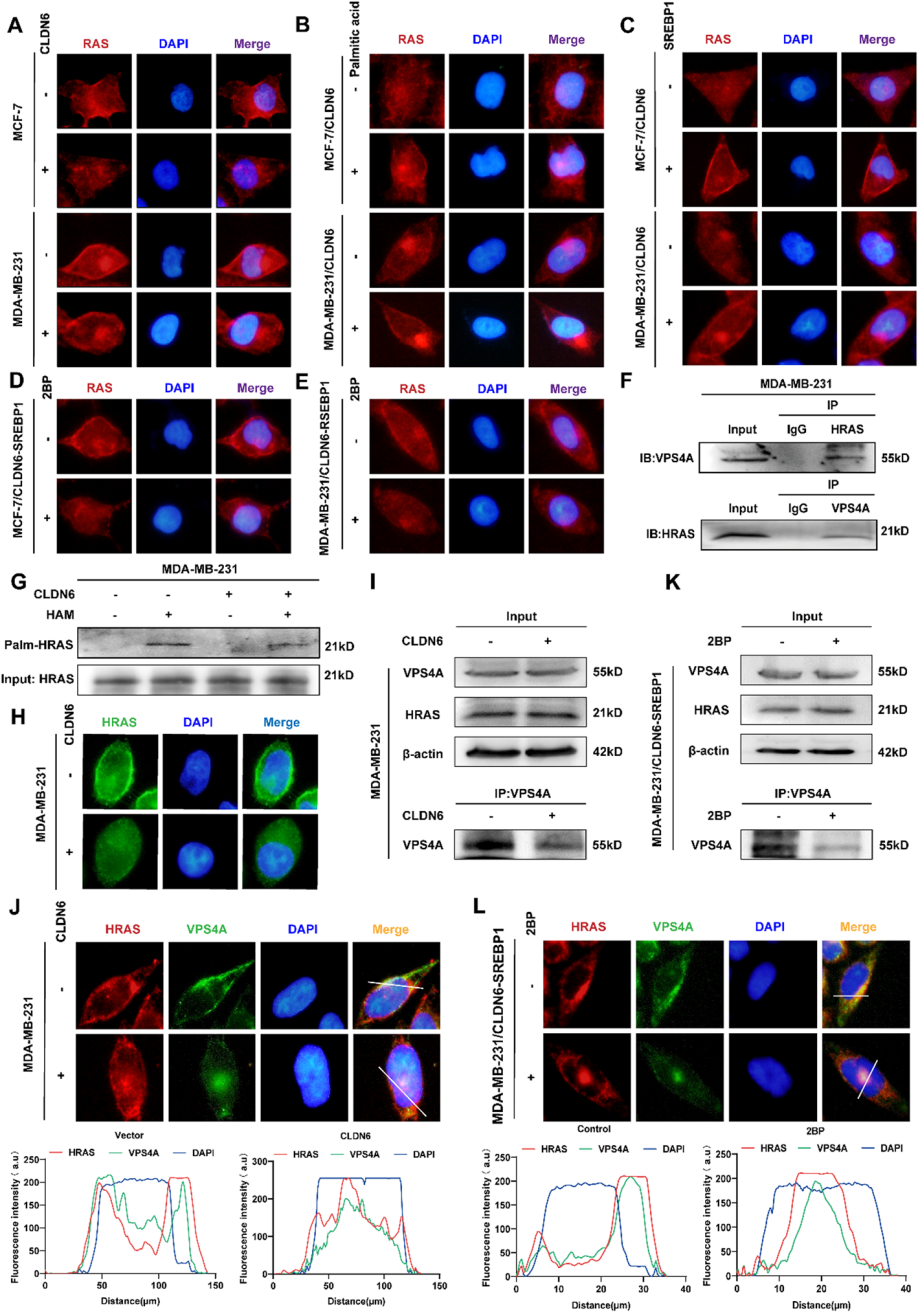
CLDN6 modulates the localization of palmitoylated RAS via VPS4A. **A**-**D** The localization of RAS (red) in BC cells with CLDN6 overexpression(**A**), palmitic acid treatment (**B**), SREBP1 overexpression (**C**), and 2BP treatment (**D**-**E**). Scale bar, 20 μm. **F** The interaction between VPS4A and HRAS. **G**, **H** The impact of CLDN6 overexpression on palmitoylation (**G**) and localization (**H**) of HRAS (green) in BC cells. Scale bar, 20 μm. **I**, **J** The regulatory impact of CLDN6 overexpression on the binding between HRAS and VPS4A (**I**) and the colocalization of HRAS (red) and VPS4A (green) (**J**). Scale bar, 20 μm.** K**-**L** The alteration of the binding between HRAS and VPS4A (**K**) and the colocalization of HRAS (red) with VPS4A (green) (**L**) in SREBP1-overexpressing BC cells induced by 2BP treatment. Scale bar, 20 μm

Since proper plasma membrane localization is crucial for RAS carcinogenic activity, IF was conducted to validate the impact of CLDN6 on RAS subcellular localization via palmitoylation. These findings indicated that overexpression of CLDN6 markedly hindered the association of RAS with the plasma membrane (Fig. 4A and Supplementary Fig 6A). Furthermore, palmitic acid treatment restored the inhibition of CLDN6 on RAS plasma membrane localization (Fig. 4B and Supplementary Fig 6B), resembling the distribution observed upon SREBP1 overexpression (Fig. 4C and Supplementary Fig 6C). Notably, the addition of 2-BP counteracted the enhanced RAS plasma membrane positioning induced by SREBP1 (Fig. 4D, E and Supplementary Fig. 7A). The above results reflected that CLDN6 impaired the RAS plasma membrane trafficking by suppressing its palmitoylation process. Consequently, we aimed to delineate the specific mechanism that CLDN6 regulated the translocation of palmitoylated RAS.

Previous reports indicated that VPS4A directly binds to HRAS, a subtype of RAS, facilitating its transportation to the plasma membrane via ESCRT [29]. In this study, we have confirmed the interaction between VPS4A and HRAS in BC cells (Fig. 4F). Consequently, we were interested in whether CLDN6 could regulate the trafficking of RAS in the same way. IP-ABE revealed that CLDN6 substantially decreased the palmitoylation of HRAS (Fig. 4G) and IF showed that CLDN6 impeded the binding of HRAS to the plasma membrane (Fig. 4H and Supplementary Fig 7B), consistent with the function of CLDN6 in modulating RAS activity. Furthermore, our findings indicated that CLDN6 attenuated the interaction between HRAS and VPS4A (Fig. 4I) and disrupted their colocalization on the plasma membrane (Fig. 4J). Similarly, the introduction of 2BP also led to a decrease in the binding of VPS4A and HRAS (Fig. 4K), resulting in the loss of their plasma membrane colocalization (Fig. 4L). These observations suggested that CLDN6 hindered the translocation of palmitoylated RAS to the plasma membrane by impairing the interaction between VPS4A and RAS.

### CLDN6 regulates SREBF1 transcription by inhibiting KLF5 nuclear translocation

**Fig. 5:**
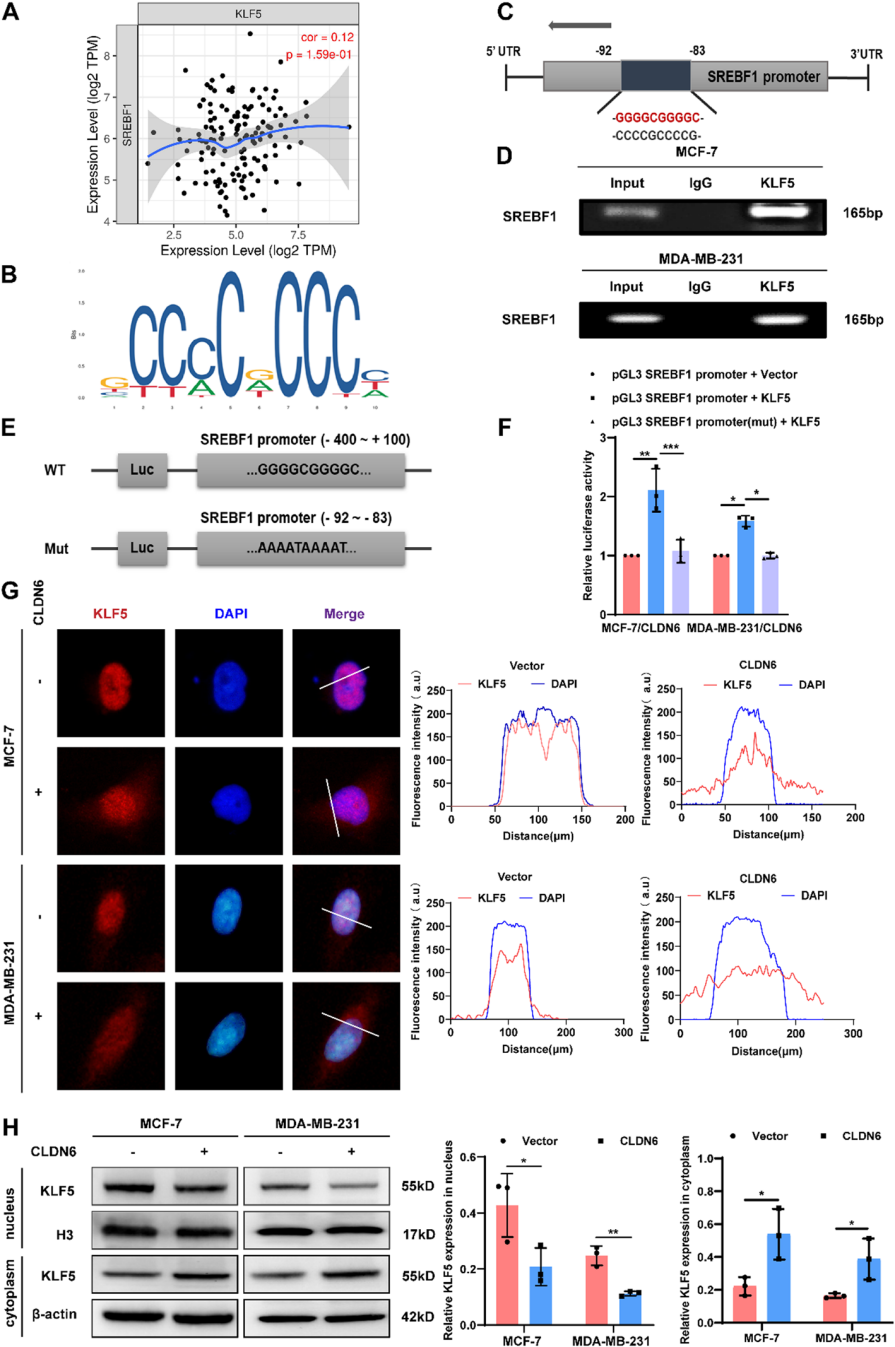
CLDN6 restrains SREBF1 transcription via modulating KLF5 nuclear translocation. **A** The SREBF1 expression in the Timer database was positively associated with the KLF5 expression in stromal BC. **B**, **C** The most likely binding site of KLF5 to the SREBF1 promoter predicted by the JASPAR database. **D** ChIP was used to detect the binding of KLF5 to SREBF1 promoter in BC cells. **E**, **F** Dual-luciferase reporter assay of BC cells transfected with WT or Mut SREBF1 promoter reporter plasmid and KLF5 plasmid (*n* = 3). **G** The impact of CLDN6 overexpression on KLF5 (red) positioning in BC cells. Scale bar, 20 μm.** H** The modulation of CLDN6 on the KLF5 nuclear and cytoplasmic distribution (*n* = 3). *P < 0.05, **P < 0.01, ***P < 0.001 denoted the presence of statistically significant disparities

The substantial decrease in SREBP1 expression suggested that CLDN6 may inhibit the transcription of SREBF1 by affecting its transcription factors. KLF5, a constituent of Krüppel-like factor family, serves as an important transcription factor regulating fatty acid synthesis [30]. Analysis from the Timer database showed a positive correlation between KLF5 and SREBF1 in stromal BC (Fig. 5A). Furthermore, JASPAR database (https://jaspar.elixir.no/) analysis demonstrated the presence of a highly probable binding site for KLF5 within the promoter region of SREBF1 (Fig. 5B, C). Therefore, ChIP assay was conducted to validate interplay of KLF5 and the promoter region of SREBF1 in BC cells (Fig. 5D). Additionally, dual luciferase reporter assay indicated relative luciferase activity of wild-type SREBF1 promoter was obviously upregulated with the overexpression of KLF5. Nevertheless, when a mutation was introduced to the binding site of KLF5 in the promoter region of SREBF1, KLF5 showed no discernible effect on the relative luciferase activity (Fig. 5E, F). These data provided compelling evidence for KLF5 as a transcription factor, which positively modulated SREBP1 expression.

Given CLDN6’s capacity to bind to transcription factors via PBM and, thus, block their nuclear translocation [31], we hypothesized that the impaired nuclear translocation of KLF5 caused by CLDN6 could potentially hinder SREBF1 transcription. IF uncovered that overexpression of CLDN6 diminished KLF5 nuclear translocation and concurrently increased its distribution in the cytoplasm and plasma membrane (Fig. 5G). Furthermore, nuclear fractionation experiments confirmed that CLDN6 effectively reduced the nuclear distribution of KLF5 while increasing its cytoplasmic distribution (Fig. 5H). Taken together, these findings strongly suggested that CLDN6 repressed SREBF1 transcription by inhibiting KLF5 nuclear translocation.

### CLDN6 recruits KLF5 by binding to MAGI2 via PDZ-binding motif

**Fig. 6:**
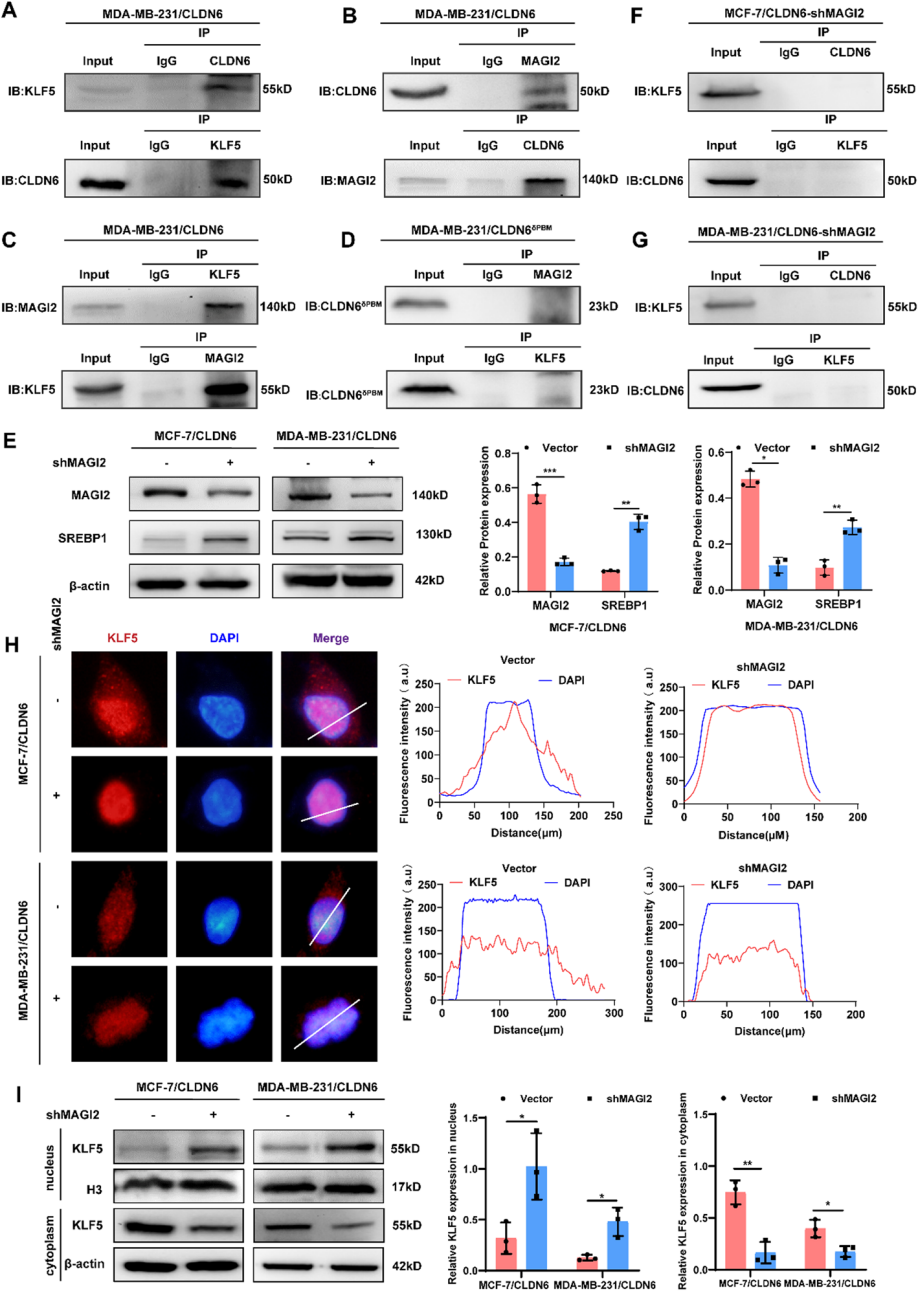
CLDN6 sequesters KLF5 in the cytoplasm through its interaction with MAGI2. **A**–**C** The interaction among CLDN6, MAGI2, and KLF5 in MDA-MB-231/CLDN6 cells. **D** The interaction between CLDN6 and MAGI2 or KLF5 in MDA-MB-231/CLDN6^δPBM^ cells. **E** The alteration of SREBP1 expression in MAGI2-knockdown BC cells (*n* = 3). **F**-**G** The interaction between CLDN6 and KLF5 in BC cells with MAGI2 knockdown. **H**, **I** The effect of MAGI2-knockdown on localization (red) (**H**) and distribution of KLF5 (**I**) (*n* = 3) in BC cells with CLDN6 overexpression. Scale bar, 20 μm. *P < 0.05, **P < 0.01, ***P < 0.001 denoted statistically significant differences

Given that CLDN6 overexpression resulted in the ectopic translocation of KLF5 from the nucleus to the cytoplasm and plasma membrane, we investigated whether this effect was due to an interaction between CLDN6 and KLF5. And the association between CLDN6 and KLF5 was confirmed by Co-IP (Fig. 6A). The result motivated us to conduct additional research into the specific mechanism of CLDN6 binding to KLF5.

MAGI2, a scaffolding protein, acts as molecular hubs for the docking of multiple proteins via its PDZ domains and WW domains. Considering the PBM of CLDN6 and the PY motif of KLF5, we postulated that MAGI2 might potentially mediate the inhibitory effect of CLDN6 on de novo fatty acid synthesis by facilitating a physical interaction between CLDN6 and KLF5. As anticipated, Co-IP verified our conjecture of the binding between MAGI2 and CLDN6 (Fig. 6B) and the interaction between MAGI2 and KLF5 (Fig. 6C). To further assess whether the interaction of CLDN6 with MAGI2 depends on PBM, the lentivirus transfection was implemented to construct a PBM mutant of CLDN6 (CLDN6^δPBM^). Here, we found that CLDN6^δPBM^ failed to interact with either KLF5 or MAGI2 (Fig. 6D). Additionally, to ascertain the impact of MAGI2 on the subcellular localization of KLF5 and the expression of SREBP1, MAGI2 was knocked down in CLDN6-overexpressing BC cells (Fig. 6E). The Co-IP results revealed that CLDN6 did not exhibit an interaction with KLF5 following MAGI2 knockdown (Fig. 6F, G). And the increased nuclear translocation (Fig. 6H) and enhanced nuclear accumulation of KLF5 (Fig. 6I) were observed. In combination, the findings suggested that CLDN6 interacted with MAGI2 through PBM to facilitate the recruitment of KLF5 and subsequently inhibited its nuclear translocation.

### CLDN6 inhibits BC growth and metastasis via palmitic acid-mediated RAS palmitoylation in vivo

**Fig. 7:**
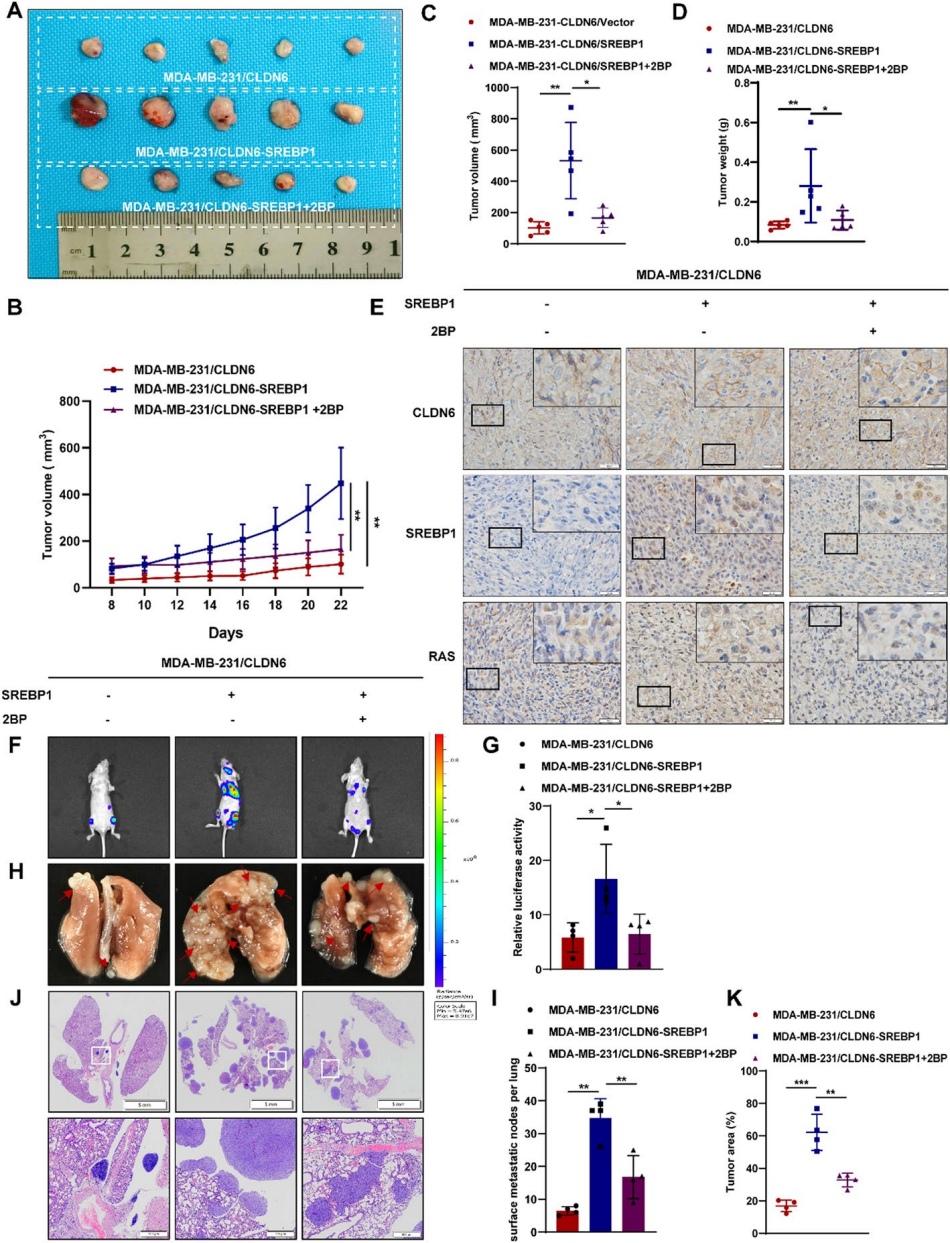
In vivo, CLDN6 inhibits BC progression through RAS palmitoylation. **A** Images of subcutaneous xenograft tumors (*n* = 5). **B**–**D** Tumor volume (**B**, **C**) and weight (**D**) of subcutaneous xenograft tumors. **E** Visualization of the expression levels and localization of CLDN6, SREBP1, and RAS in transplanted tumor tissue. Scale bar, 20 μm. **F**, **G** Analysis of lung metastasis using bioluminescent techniques (*n* = 4). **H**, **I** Lung metastatic nodes were indicated with red arrowheads. **J**, **K** The lung metastasis of BC was observed through H&E staining. Scale bar, 5 mm and 500 μm. *P < 0.05, **P < 0.01, ***P < 0.001 denoted statistically significant differences

In our prior investigation, it was uncovered that the growth and metastasis of BC were impeded by CLDN6 in vivo. However, the precise biological function of CLDN6-modulated RAS palmitoylation through palmitic acid remains elusive. Herein, we established a nude mouse xenograft model and divided the experimental groups into three: MDA-MB-231/CLDN6, MDA-MB-231/CLDN6-SREBP1 and MDA-MB-231/CLDN6-SREBP1 + 2BP. The results showed that tumor growth rate and weights of MDA-MB-231/CLDN6-SREBP1 group increased considerably versus the MDA-MB-231/CLDN6 group. In line with pharmacologic inhibition of RAS palmitoylation in vitro, treatment with 2BP noticeably restored the enhancement induced by SREBP1 on tumor growth rate and weights (Fig. 7A–D). In addition, IHC demonstrated that SREBP1 effectively counteracted the disruptive impact of CLDN6 on RAS cell membrane localization, while 2BP abolished the promotional effect of SREBP1 (Fig. 7E). These findings indirectly suggested that SREBP1 reversed the inhibitory impact of CLDN6 on RAS palmitoylation in tumor tissues, while the palmitoylation inhibitor 2BP collaborated with CLDN6 to suppress RAS palmitoylation. And, in line with our earlier observation in vitro, overexpression of SREBP1 led to a prominent increase in neutral lipids in transplanted tumor tissues (Supplementary Fig. 8A).

Meanwhile, to validate the inhibitory effect of CLDN6 on BC metastasis via palmitic acid-mediated RAS palmitoylation, we established a BC lung metastasis model. The occurrence of tumor metastasis was assessed through luciferase imaging (Fig. 7F, G) and macroscopic observations (Fig. 7H, I). Nude mice that received injections of MDA-MB-231/CLDN6-SREBP1 cells demonstrated an increased incidence of lung metastasis in comparison with the control group. However, this trend was reversed when the mice were treated with 2BP. H&E staining confirmed that overexpression of SREBP1 counteracted the suppressive effect of CLDN6 on lung metastasis, whereas addition of 2BP led to a reduction in the area of lung metastasis (Fig. 7 J, K). Taken together, these results strongly suggested that CLDN6 restrained palmitoylation and cell membrane translocation of RAS by reducing SREBP1-mediated de novo palmitic acid synthesis to inhibit BC progression.

### The clinical association among the expression of CLDN6, SREBP1, and RAS in patients with BC

**Fig. 8:**
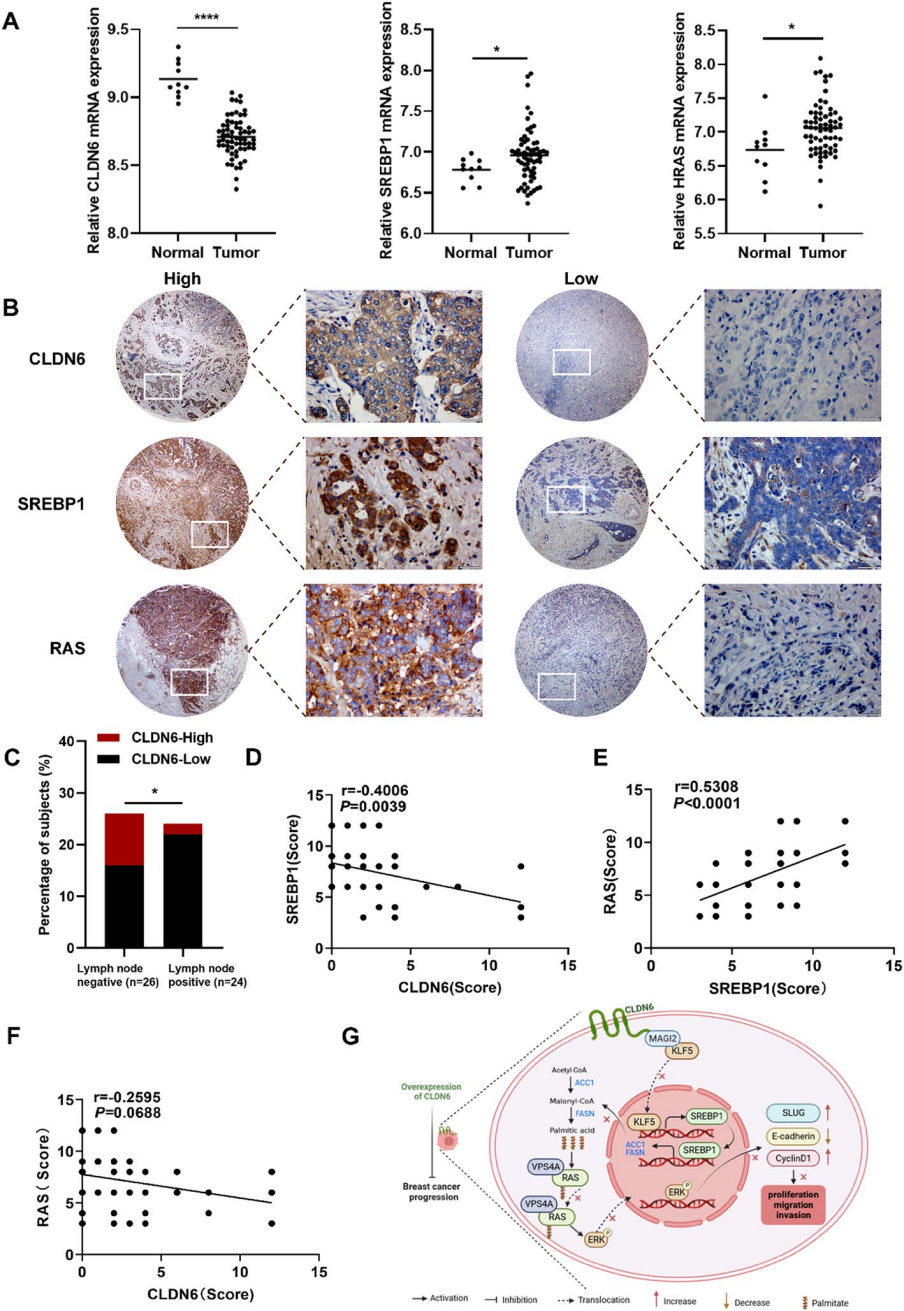
Clinical association among the expression of CLDN6, SREBP1, and RAS in patients with BC. **A** The levels of CLDN6, SREBP1, and HRAS mRNA expression were compared between normal tissues and BC tissues using GSE103512 (normal, 10 and tumor, 65).** B** Visualization of the low and high expression of CLDN6, SREBP1 and RAS in human BC tissues (*n* = 50). Scale bar, 200 μm (left) and 20 μm (right). **C** The distribution of CLDN6 expression levels in patients with BC with and without lymph node involvement. **D**–**F** Correlation analysis was conducted on a BC tissue microarray, examining the relationship between CLDN6 and SREBP1 (**D**), SREBP1 and RAS (**E**), as well as CLDN6 and RAS (**F**) (*n* = 50). **G** A suggested framework elucidating the regulatory process by which CLDN6 hinders the progression of BC via SREBP1-facilitated RAS palmitoylation. *P < 0.05, **P < 0.01, ***P < 0.001 denoted statistically significant differences

To validate the expression of CLDN6, SREBP1, and RAS in BC, we conducted an analysis on the GSE103512 dataset from the GEO database. The findings indicated a downregulation of CLDN6 in BC tissues compared with normal tissues, while SREBP1 and HRAS exhibited upregulated expression levels relative to normal tissues (Fig. 8A).

Subsequently, we aimed to determine the clinical significance of the CLDN6-regulated RAS palmitoylation and assess the relationship between CLDN6, SREBP1, RAS and clinicopathological parameters. To achieve this, we performed IHC in BC tissue microarrays from 50 patients (Fig. 8B). As depicted in Table 1, we observed that patients with low CLDN6 expression exhibited a higher likelihood of lymph node metastasis (Fig. 8C). However, no substantial correlation was observed between the expression of SREBP1 or RAS and clinical characteristics, possibly due to the small size of the sample. The analysis of Pearson correlation indicated an inverse relationship between the expression of CLDN6 and SREBP1 in clinical BC tissues (Fig. 8D), while SREBP1 expression exhibited a positive correlation with RAS expression (Fig. 8E). Nonetheless, there was no noticeable association found between the expression of CLDN6 and RAS (Fig. 8F).

**Table 1:** The correlation among CLDN6, SREBP1, and RAS expression and clinical features of patients with BC

Clinicopathologic features	CLDN6			SREBP1			RAS		
	Low	High	*P*	Low	High	*P*	Low	High	*P*
Age									
≤ 57 years	18	5	0.73	12	11	0.179	12	11	0.283
> 57 years	20	7		9	18		10	17	
Tumor size									
≤ 3 cm	23	7	0.892	13	17	0.815	15	15	0.295
> 3 cm	15	5		8	12		7	13	
Pathology grade									
I–II	18	5	0.73	8	15	0.34	11	12	0.615
II–III	20	7		13	14		11	16	
Lymph node									
Negative	16	10	0.013*	12	14	0.536	12	14	0.749
Positive	22	2		9	15		10	14	
Tumor stage									
0–IIA	19	7	0.614	11	15	0.963	10	16	0.412
IIB–III	19	5		10	14		12	12	

*n* = 50, *P < 0.05

## Discussion

In this study, we provide a novel theory proposing that impeding SREBP1-mediated RAS palmitoylation with CLDN6 serves as a promising therapeutic strategy to restrain BC progression. Our previous investigations have demonstrated that CLDN6 is downregulated in BC and the overexpression of CLDN6 inhibits BC progression. Here, we observed that CLDN6 suppressed the palmitic acid-induced RAS palmitoylation through the MAGI2/KLF5/SREBP1 axis, thereby hindering BC malignant progression.

As an integral part of the tight junction proteins family, CLDN6 is crucial for maintaining cellular integrity and permeability. Recently, CLDN6 has garnered increasing attention due to its modulation of intracellular signal transduction. Our prior research has observed the involvement of CLDN6 in fatty acid anabolism. Nevertheless, the impact of CLDN6 on fatty acid metabolism and its specific mechanism remain fully elucidated. In current research, we observed that CLDN6 participated in lipid homeostasis regulation within BC cells. Overexpression of CLDN6 significantly reduced the synthesis of palmitic acid along with the generation of triglyceride and neutral lipid. In addition, supplementation with exogenous palmitic acid effectively reversed the suppressive impact of CLDN6 on BC cells proliferation, migration, and invasion. These findings highlight the significant involvement of CLDN6 in fatty acid anabolism, which regulates BC malignant phenotype.

In contrast to normal cells, tumor cells predominantly undergo endogenously de novo fatty acids synthesis [32]. As the central switch of intracellular lipid synthesis, SREBP1 transcriptionally modulates the expression of lipogenic enzymes to regulate intracellular fatty acid content [33, 34]. The expression of SREBP1 is regulated by transcription factors, classical carcinogenic signaling pathways and protein kinases [35]. Our investigation revealed that CLDN6 downregulated the expression of SREBP1 by inhibiting the nuclear accumulation of KLF5, a well-known master transcription factor containing the PY motif. We identified and reported for the first time the precise binding site of KLF5 within SREBF1 promoter region. Mechanistically, CLDN6 bound to MAGI2 through PBM then utilized the WW domain of MAGI2 to interact with KLF5, thereby hindering the nuclear translocation of KLF5. However, the recruitment effect of CLDN6 on KLF5 was highly dependent on PBM, and this effect was abolished upon either PBM mutation or knockdown of MAGI2. Importantly, further investigations are warranted to identify the specific WW domain within MAGI2 that binds to KLF5, potentially offering a novel target for regulating tumor fatty acid synthesis.

Palmitoylation is an important post-transcriptional modification that affects the subcellular localization and carcinogenic activity of multiple oncogenes. Research has demonstrated that RAS promotes its carcinogenic activation through more plasma membrane distribution. Palmitoylation facilitates the utilization of palmitic acid by RAS to anchor itself to the phospholipid bilayer, thereby ensuring efficient transmission and release of signals. We demonstrated that CLDN6 inhibited RAS palmitoylation by reducing the synthesis of substrate palmitic acid. Increasing endogenous palmitic acid by SREBP1 or adding exogenous palmitic acid could reverse the inhibitory effect of CLDN6 on RAS palmitoylation. Additionally, the addition of 2BP reversed the enhancement of RAS carcinogenic activity induced by endogenous palmitic acid, inducing a significant inhibition of RAS signaling pathway activity. On the other hand, CLDN6 manipulates the correct plasma membrane localization necessary for the palmitoylated RAS. It has been shown that HRAS exhibited high expression in BC tissues [36, 37]. Abnormal activation of HRAS, rather than NRAS, induced malignant transformation of human breast epithelial cells [38, 39]. Here, we revealed that CLDN6 induced a decrease in the binding of palmitoylated HRAS to VPS4A, thereby preventing palmitoylated HRAS from being transported to plasma membrane through the ESCRT. These results verify that CLDN6 relies on palmitic acid to effectively suppress the carcinogenic activation of RAS at different levels, thereby hindering the RAS-driven BC malignant progression.

## Conclusions

In summary, we present a viable interpretation that CLDN6 represses palmitic acid biosynthesis to impede the BC progression attributed to RAS palmitoylation (Fig. 8G). This study offers an innovative insight that monitoring CLDN6 expression and implementing targeted interventions of palmitic acid synthesis is an effective strategy for patients with BC with RAS oncogenic activation.

### Supplementary Information

Additional file 1.

## Data Availability

The datasets used and/or analyzed during the current study are available from the corresponding author on reasonable request.

## Abbreviations

BC: Breast cancer
CCK-8: Cell counting kit 8 assay
ChIP: Chromatin immunoprecipitation assay
Co-IP: Coimmunoprecipitation assay
ESCRT: Endosomal sorting complex required for transport
IF: Immunofluorescence
IHC: Immunohistochemistry
IP-ABE: Immunoprecipitation and acyl–biotin exchange
PBM: PDZ-binding motif
RT–PCR: Reverse transcription polymerase chain reaction
