# Supplementary material for: CLDN6 inhibits breast cancer growth and metastasis through SREBP1-mediated RAS palmitoylation
Source: Cell Mol Biol Lett. 2024 Aug 21;29:112. doi: 10.1186/s11658-024-00629-y (PMC11337767; doi:10.1186/s11658-024-00629-y)
Supplement: Supplementary file 1 — Additional file 1. [file 11658_2024_629_MOESM1_ESM.docx]

**Supplementary Materials**

**CLDN6 inhibits breast cancer growth and metastasis through SREBP1-mediated RAS palmitoylation**

Qiu Jin^1^, Da Qi^1^, Mingzi Zhang^2^, Huinan Qu^3^, Yuan Dong^1^, Minghao Sun^1^, Chengshi Quan^1^ *

* Corresponding Author

^1^ The Key Laboratory of Pathobiology, Ministry of Education, College of Basic Medical Sciences, Jilin University, 126 Xinmin Avenue, Changchun, 130021, Jilin, China.

^2^ The Zilkha Neurogenetic Institute, Department of Physiology and Neuroscience Keck School of Medicine of the University of Southern California, 1501 San Pablo street, Los Angeles, 90033, California, US.

^3^ Department of Histology and Embryology, College of Basic Medical Sciences, Jilin University, 126 Xinmin Avenue, Changchun, 130021, Jilin, China.

E-mails: [quancs@jlu.edu.cn](mailto:quancs@jlu.edu.cn)


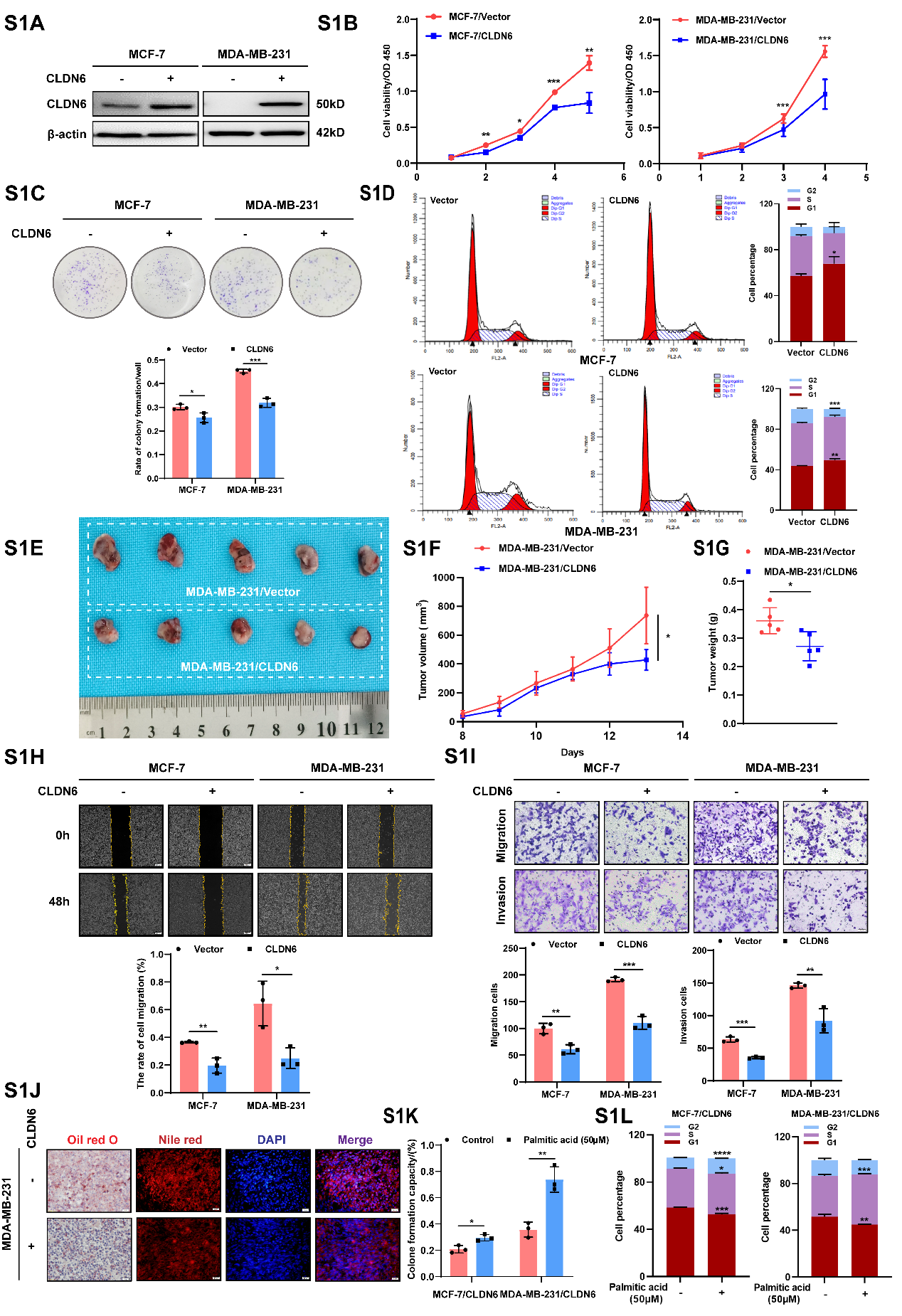


**Supplementary Fig. 1 CLDN6 inhibits breast cancer progression.**

**S1A**: Identification of CLDN6 overexpression in MCF-7 and MDA-MB-231 cells. **S1B-D**: The impact of CLDN6 overexpression on cell viability (**S1B**), clonogenicity (**S1C**) and cell cycle progression (**S1D**) of BC cells. **S1E-G**: Images of subcutaneous xenograft tumors (**S1E**) (n=5). **S1F-G**: Tumor volume (**S1F**) and weight (**S1G**) of subcutaneous xenograft tumors. **S1H-I**: Wound healing assay (**S1H**) and transwell migration and invasion assays (**S1I**) were performed in CLDN6-overexpression cells. Scale bar: 200 μm (**S1H**) and 50 μm (**S1I**). (n=3) **S1J**：Oil red O staining and Nile red staining of neutral lipids in xenograft tumor tissues. Scale bar: 20 μm. **S1K**: The effect of palmitic acid on the clonogenicity of MCF-7/CLDN6 and MDA-MB-231/CLDN6 (n=3). **S1L**: The alteration of cell cycle progression with or without palmitic acid. **P*<0.05, ***P*<0.01, ****P*<0.001.


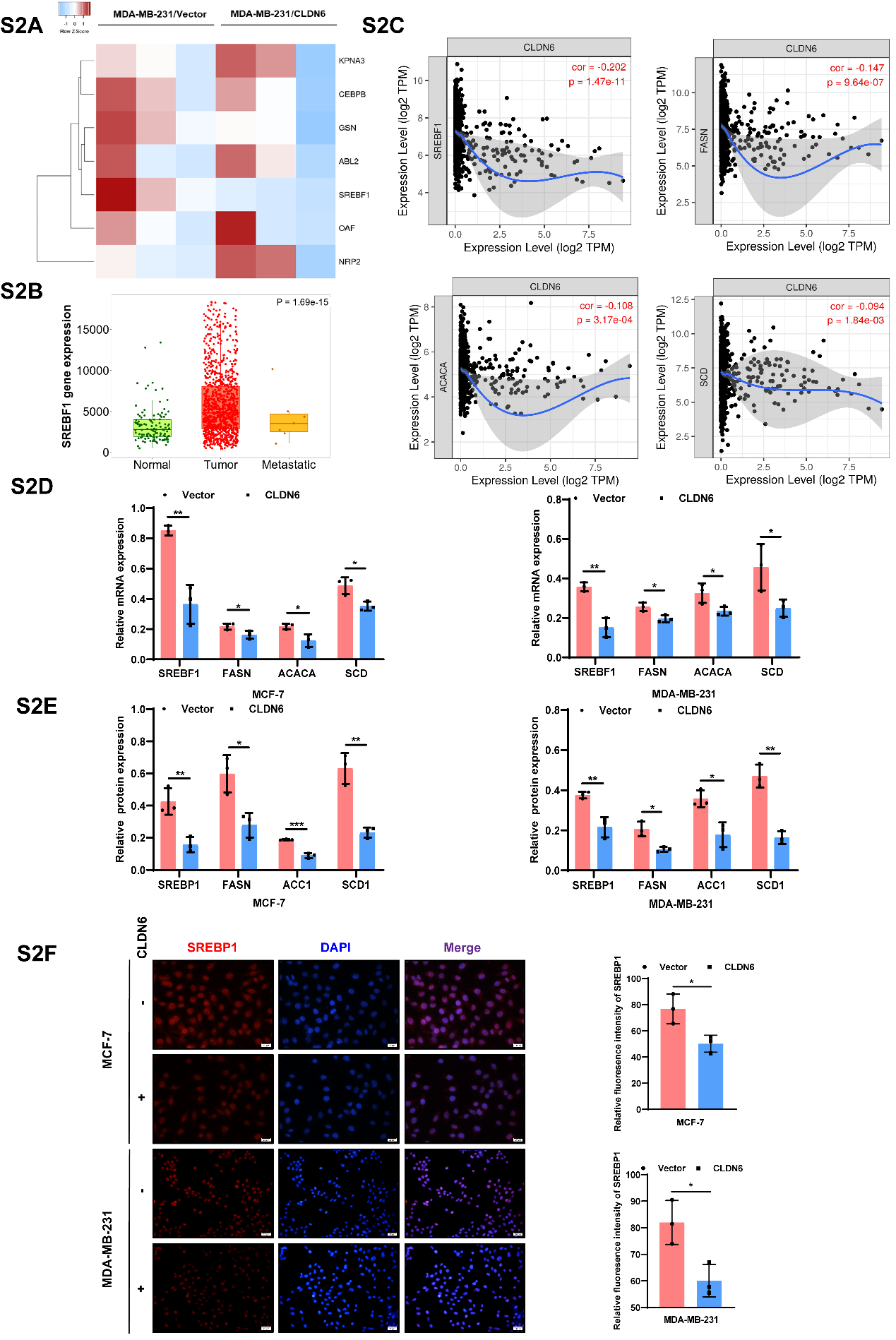
**Supplementary Fig. 2:** **CLDN6 inhibits the expression of SREBP1, FASN, ACC1 and SCD1 at both mRNA and protein levels.**

**S2A**: Heatmap showed the genes affected by CLDN6 overexpression in MDA-MB-231 cells. **S2B**: The expression of SREBF1 was significantly increased in primary and metastatic tumor tissues compared with normal tissues. **S2C**: The expression of SREBF1, FASN, ACACA and SCD was negatively correlated with the expression of CLDN6 in BC. **S2D-E**: The effect of CLDN6 on SREBF1, FASN, ACACA and SCD expression at mRNA and protein levels in BC cells. **S2F**: The SREBP1 expression was detected by IF in BC cells with CLDN6 overexpression. **P*<0.05, ***P*<0.01, ****P*<0.001.


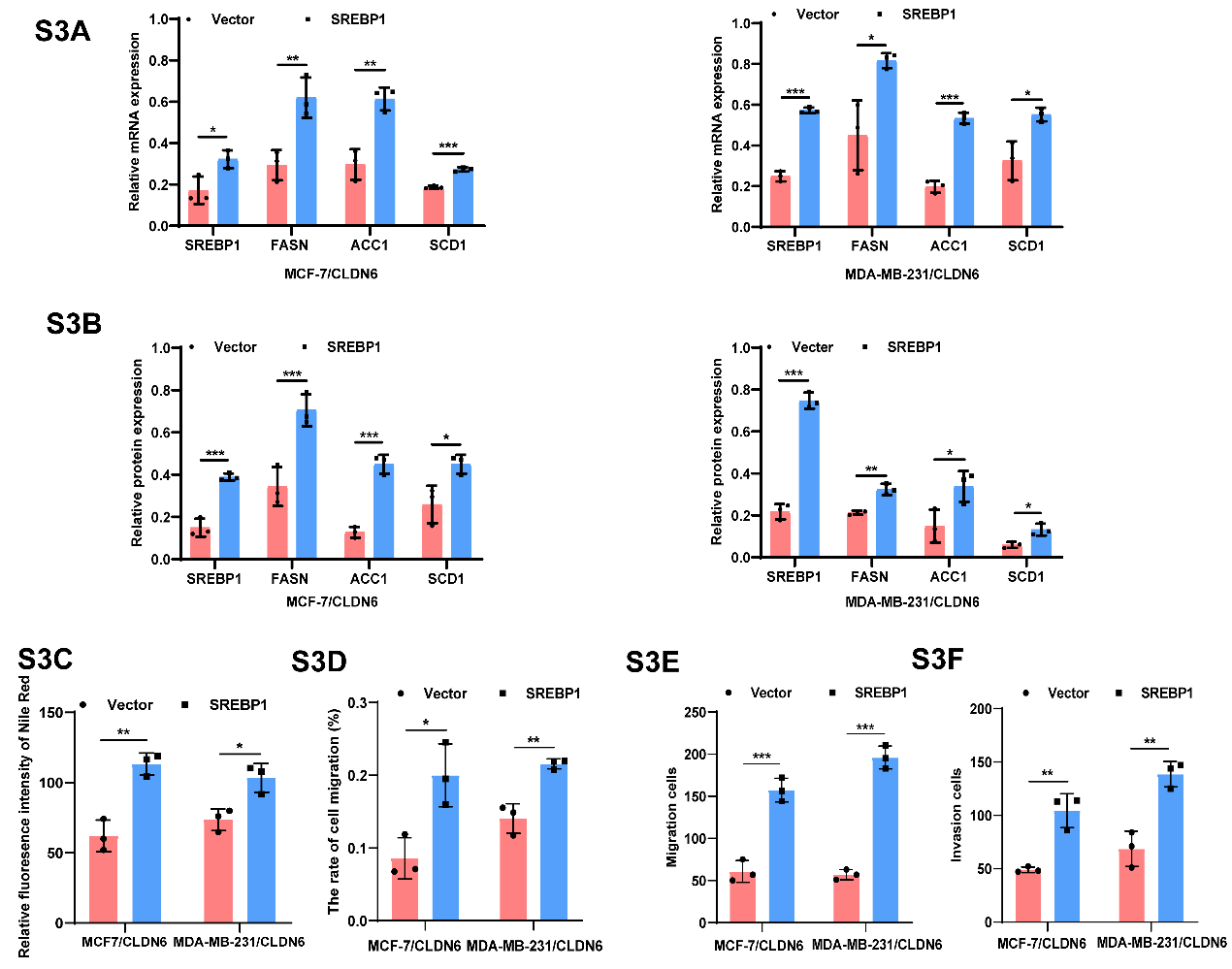


**Supplementary Fig. 3:** **CLDN6 inhibits *de novo* fatty acid synthesis through SREBP1.**

**S3A**: RT-PCR was utilized to assess the efficacy of SREBP1 overexpression and its impact on the expression of FASN, ACACA, and SCD. **S3B**: Western blot analysis was employed to assess the efficacy of SREBP1 overexpression and its impact on the expression of FASN, ACC1, and SCD1. **S3C**: Statistical analysis of relative fluorescence intensity of Nile red in BC cells overexpressing CLDN6. **S3D-F:** Statistical analysis of wound healing assay and transwell migration and invasion assays of SREBP1-overexpressing BC cells. **P*<0.05, ***P*<0.01, ****P*<0.001.


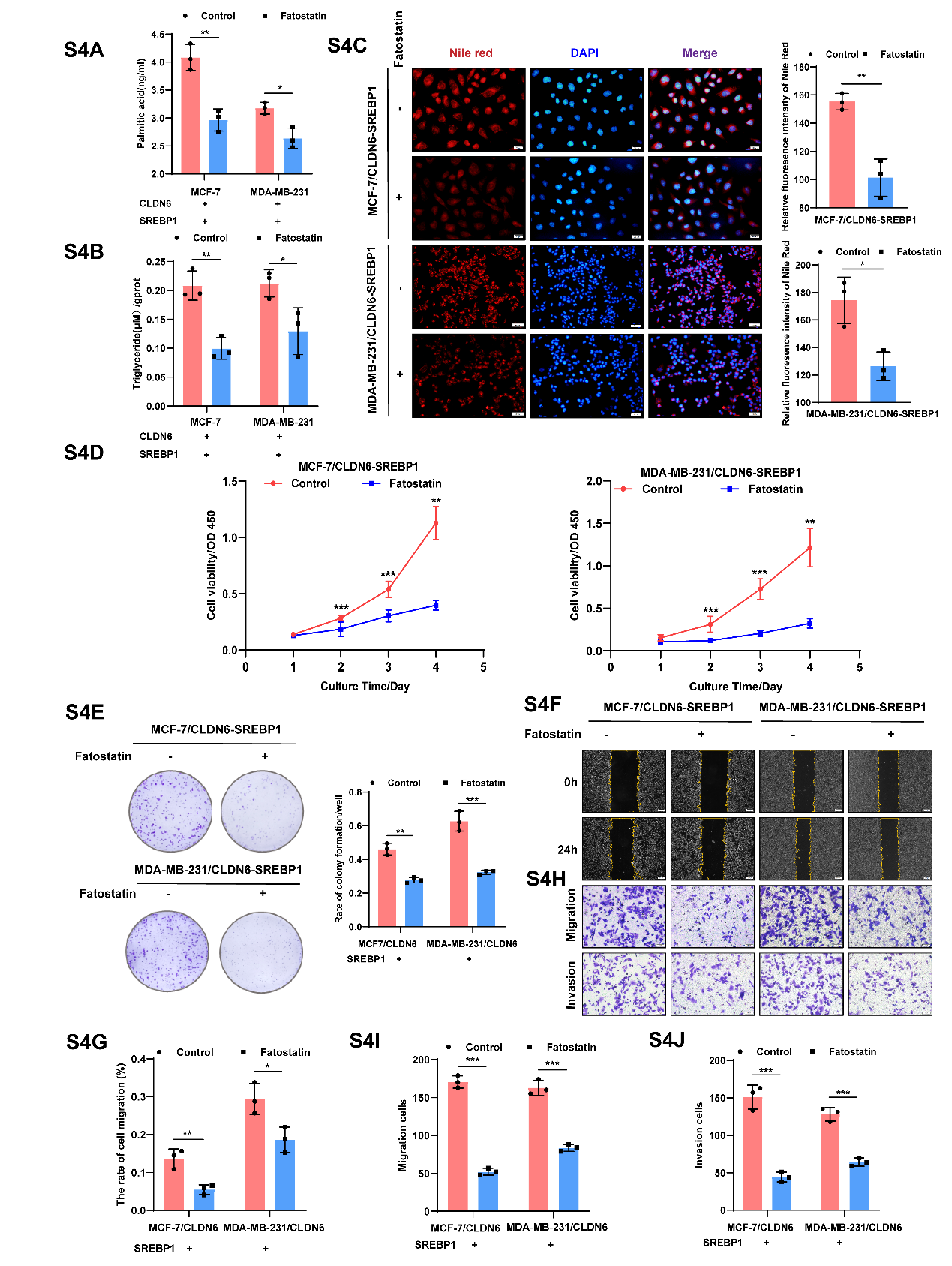


**Supplementary Fig. 4: Fatostatin inhibits the proliferation, migration and invasion promoted by SREBP1-mediated *de novo* fatty acid synthesis**

**S4A-C**: Effects of Fatostatin on palmitic acid (**S4A**), triglyceride (**S4B**) and neutral lipid **(S4C)** of MCF-7/CLDN6-SREBP1 and MDA-MB-231/CLDN6-SREBP1 cells. Scale bar: 20/50 μm. **S4D-J**: The effect of Fatostatin on cell viability (**S4D**), clonogenicity (**S4E),** migratory and invasive capacity (**S4F-J**) of MCF-7/CLDN6-SREBP1 and MDA-MB-231/CLDN6-SREBP1 cells. Scale bar: 200 μm (**F**) and 50 μm (**H**). **P*<0.05, ***P*<0.01, ****P*<0.001.


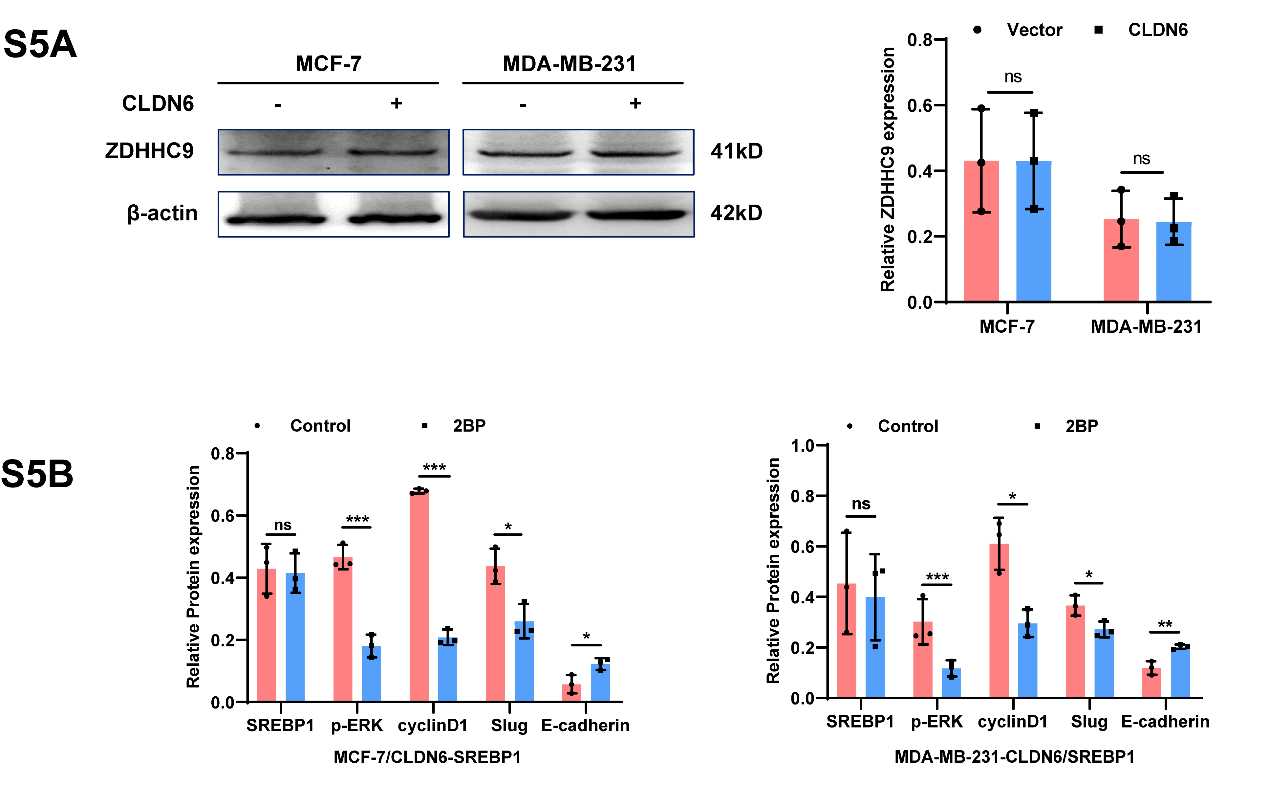


**Supplementary Fig. 5:** **CLDN6 governs palmitic acid synthesis to suppress RAS palmitoylation**

**S5A**: The expression of ZDHHC9 in BC cells with CLDN6 overexpression. ns: no significance. **S5B**: Quantification of the protein expression in RAS/ERK signaling pathway.


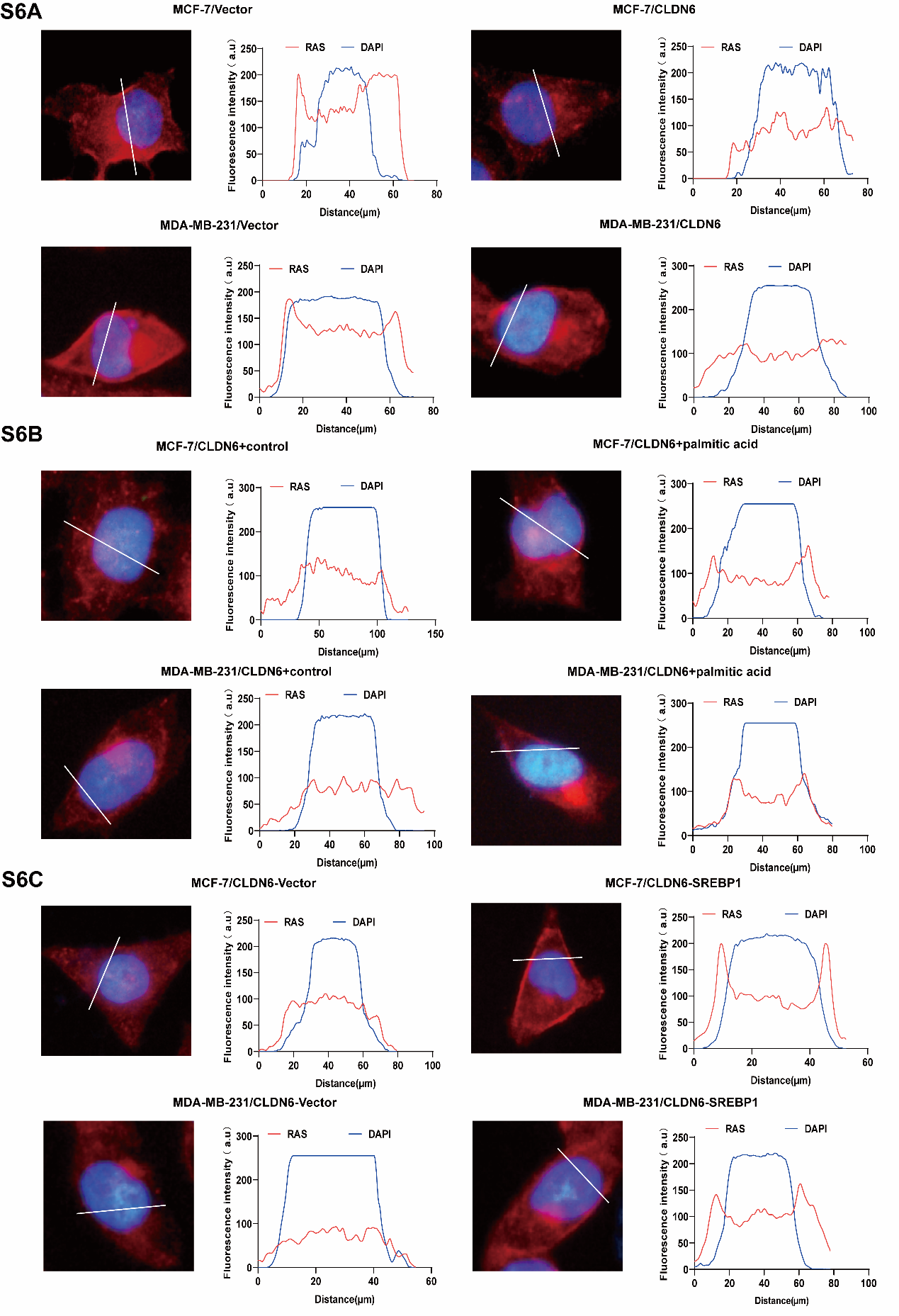


**Supplementary Fig. 6:** **CLDN6 relies on SREBP1 to** **modulate the** **plasma membrane localization of RAS**

**S6A-C**: The localization of RAS (red) in BC cells with CLDN6 overexpression(**A**), palmitic acid treatment (**B**), SREBP1 overexpression (**C**). Scale bar: 20 μm.


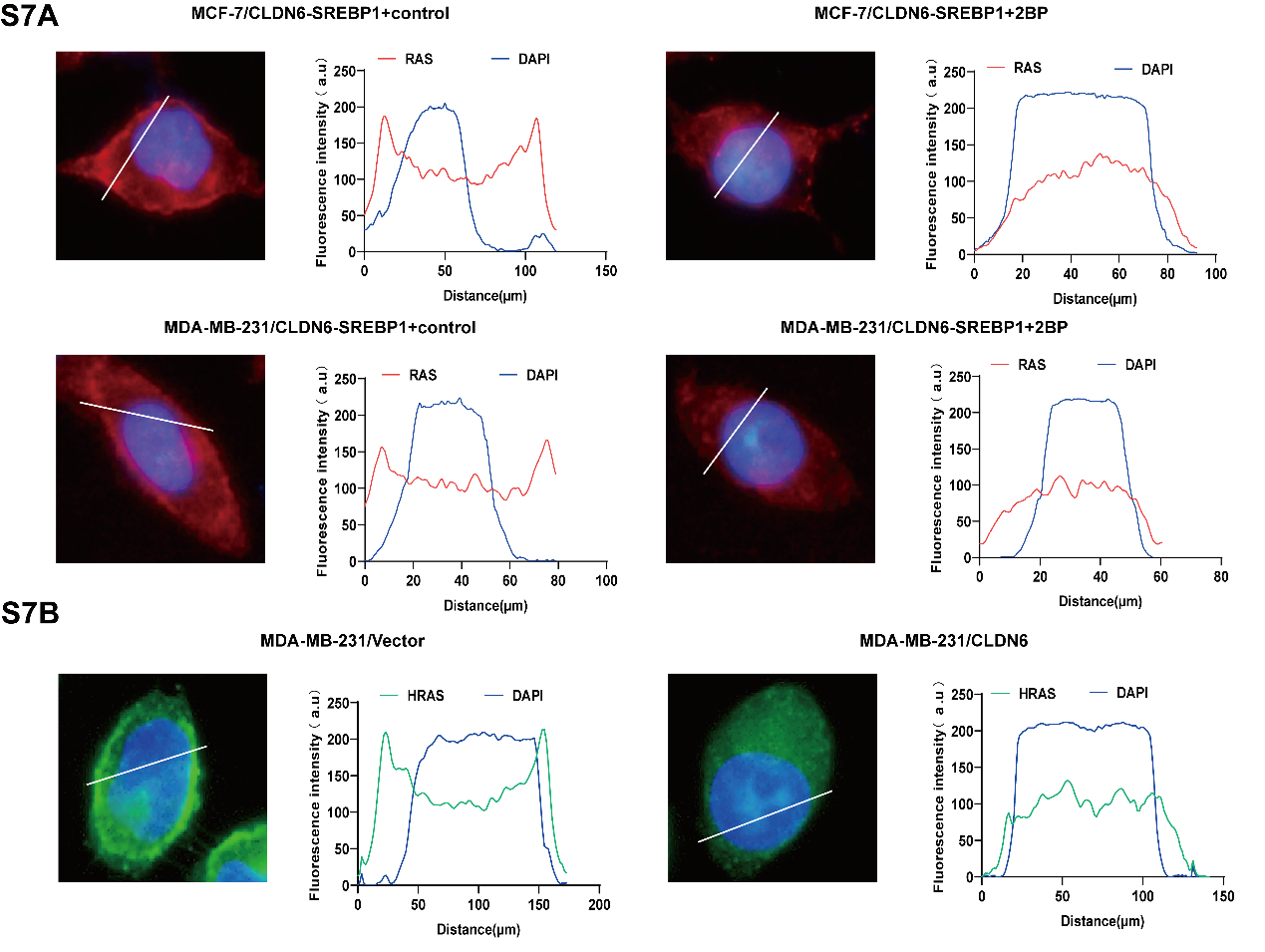


**Supplementary Fig. 7:** **The plasma membrane localization of RAS depends on the regulation of CLDN6 on SREBP1**

**S7A**: The localization of RAS (red) in BC cells with 2BP treatment. **S7B**: Effect of CLDN6 overexpression on localization of HRAS (green) in BC cells. Scale bar: 20 μm.


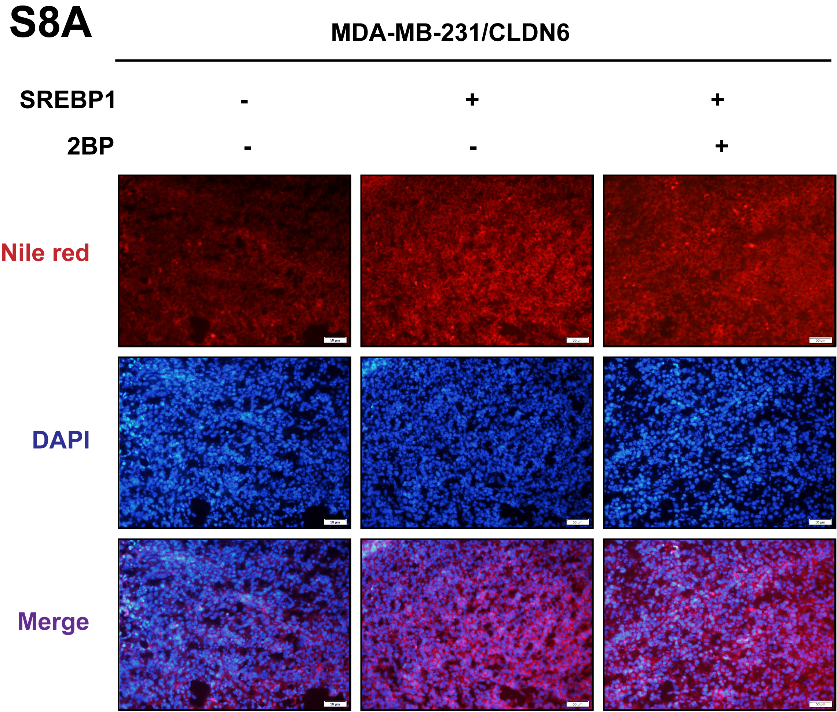


**Supplementary Fig. 8: The neutral lipid content in xenograft tumor tissues**

**S8A**: Nile red staining of the effects of SREBP1 overexpression as well as 2BP treatment on the neutral lipid content in xenograft tumor tissues. Scale bar: 50 μm.

**Supplementary Table 1 Antibodies utilized in this study.**

| **Antibody** | **Application** | **Source** | **Catalog number** |
| --- | --- | --- | --- |
| CLDN6  SREBP1 | WB/IHC/IP  WB/IHC/IF | Santa Cruz  Proteintech | sc-393671  14088-1-AP |
| FASN  ACC1 | WB  WB | Zenbio  Proteintech | R381582  21923-1-AP |
| SCD1 | WB | Proteintech | 28678-1-AP |
| SCD1 | WB | Zenbio | R25675 |
| MAGI2 | WB/IP | Santa Cruz | sc-25664 |
| β-actin  KLF5  RAS  HRAS | WB  chip/WB/IF/IP  IP-ABE/IF/IHC  IF  WB/IP/IP-ABE | Proteintech  Proteintech  Abcam  Santa Cruz  Proteintech | 66009-1-Ig  21017-1-AP  ab52939  sc-29  18295-1-AP |
| VPS4A | IP/IF | Santa Cruz | sc-393428 |
| ERK | WB | Zenbio | 343830 |
| p-ERK | WB | Zenbio | R380698 |
| CyclinD1 | WB | Proteintech | 60186-1-Ig |
| SLUG | WB | Abcam | ab183760 |
| E-cadherin | WB | Proteintech | 20874-1-AP |
| GAPDH | WB | Proteintech | 60004-1-Ig |
| ZDHHC9 | WB | Proteintech | 24046-1-AP |
| H3 | WB | Proteintech | 17168-1-AP |

**Note:** **The primary antibodies used for CLDN6, MAGI2, KLF5, HRAS, and VPS4A in Western blot and co-IP experiments are identical**

**Supplementary Table 2 Primers utilized in this study.**

| **For RT-PCR** |  |
| --- | --- |
| CLDN6  SREBF1  β-actin | CTGCCCATGTGGAAGGTGAC  GGTAGACCAGCAAGCCGAAC  CGGAACCATCTTGGCAACAGT  CGCTTCTCAATGGCGTTGT  TCATGAAGTGTGACGTGGACATC |
| FASN  ACACA  SCD | CAGGAGGAGCAATGATCTTGATCT  TCCACCAAGTCCAACATGGG  GGGCTATGGAAGTGCAGGTT  CTCTTGGCCTTTTCCCGGTC  GCCTCAATTTCCCTTGCTGC  GGAGCCACCGCTCTTACAAA  GAAAACTTGTGGTGGGCACG |
| **For CHIP-PCR** |  |
| SREBF1 | CTGAGGGGAGATTTGTGGCG |
|  | AAGTTCCTCGGAAACTGGGTT |

**Supplementary Table 3 Reagents utilized in this study.**

| **The reagents** | **Source** | **Code** | **Working**  **concentrations** | **Treatment time** |
| --- | --- | --- | --- | --- |
| Fatostatin | MCE | HY-14452 | 48μM | 24h |
| 2-bromopalmitate (2BP) | Selleck | E0120 | 100μM | 24h |
| Palmitic acid  C75 | Kunchuang biotechnology  MCE | SYSJ-KJ003  HY-12364 | 50μM  30μM | 24h  24h |
